# ProcCluster^®^ and procaine hydrochloride inhibit the replication of influenza A virus *in vitro*

**DOI:** 10.3389/fmicb.2024.1422651

**Published:** 2024-08-14

**Authors:** Clio Häring, Josefine Schroeder, Johannes Jungwirth, Bettina Löffler, Andreas Henke, Beatrice Engert, Christina Ehrhardt

**Affiliations:** ^1^Section of Experimental Virology, Institute of Medical Microbiology, Center for Molecular Biomedicine (CMB), Jena University Hospital, Jena, Germany; ^2^Institute of Medical Microbiology, Jena University Hospital, Jena, Germany; ^3^inflamed pharma GmbH, Jena, Germany

**Keywords:** local anesthetics, procaine, influenza A virus, antivirals, host-directed therapy

## Abstract

**Introduction:**

Treatment of influenza A virus infections is currently limited to few direct acting antiviral substances. Repurposing other established pharmaceuticals as antivirals could aid in improving treatment options.

**Methods:**

This study investigates the antiviral properties of ProcCluster^®^ and procaine hydrochloride, two derivatives of the local anesthetic procaine, in influenza A virus infection of A549, Calu-3 and MDCK cells.

**Results:**

Both substances inhibit replication in all three of these cell lines in multi-cycle experiments. However, cell line-dependent differences in the effects of the substances on viral RNA replication and subsequent protein synthesis, as well as release of progeny viruses in single-cycle experiments can be observed. Both ProcCluster^®^ and procaine hydrochloride delay endosome fusion of the virus early in the replication cycle, possibly due to the alkaline nature of the active component procaine. In A549 and Calu-3 cells an additional effect of the substances can be observed at late stages in the first replication cycle. Interestingly, this effect is absent in MDCK cells. We demonstrate that ProcCluster^®^ and procaine hydrochloride inhibit phospholipase A_2_ (PLA_2_) enzymes from A549 but not MDCK cells and confirm that specific inhibition of calcium independent PLA_2_ but not cytosolic PLA_2_ has antiviral effects.

**Discussion:**

We show that ProcCluster^®^ and procaine hydrochloride inhibit influenza A virus infection at several stages of the replication cycle and have potential as antiviral substances.

## 1 Introduction

Respiratory infections with influenza A virus (IAV) are a major cause of morbidity and mortality around the world (Paget et al., 2019). Treatment options for severe influenza virus infections are currently limited to a few direct-acting antiviral substances such as oseltamivir. Development of alternative treatment options is therefore of importance. Host-directed therapy aims at inhibiting IAV replication by modulating host factors on which the virus depends for replication.

To infect the host cell the IAV attaches to the cell via binding of the viral hemagglutinin (HA) to N-acetylneuraminic acid (sialic acid) residues of glycoproteins and glycolipids on the cell surface and the virus enters the cell via clathrin-dependent or clathrin-independent endocytosis (Weis et al., 1988; Fujioka et al., 2011; Air, 2014; Byrd-Leotis et al., 2017; Shi et al., 2021). After the virus is taken up by the cell, increasing acidification during the progression from the early endosome to the late endosomal compartment and proton influx through the viral matrix protein 2 (M2) ion channel cause a conformational change in the viral HA that allows fusion of the viral membrane with the endosomal membrane and the release of the viral ribonucleoproteins (vRNPs) into the cytoplasm (Yoshimura and Ohnishi, 1984; Bullough et al., 1994; White and Whittaker, 2016; Dou et al., 2018). These are then imported into the nucleus for transcription and replication by the IAV RNA-dependent RNA polymerase (RdRp) (Hutchinson and Fodor, 2012). The viral messenger RNA (mRNA) is exported from the nucleus and translated by cytosolic and endoplasmic reticulum (ER)-associated ribosomes. Newly synthesized nucleoproteins (NP) as well as the components of the RdRp subsequently form vRNPs with the newly replicated viral RNA. The vRNPs then have to travel to the plasma membrane for the assembly of new virus particles (O’Neill et al., 1998; Neumann et al., 2000). The viral membrane proteins HA, neuraminidase (NA) and M2 move to the membrane via the ER and Golgi apparatus (Dou et al., 2018). The budding process, which appears to take place at raft sites in the plasma membrane (Takeda et al., 2003; Nayak et al., 2009; Veit and Thaa, 2011), requires the induction of membrane curvature as the IAV gains its envelope by bending it out of the plasma membrane. Membrane curvature is presumed to be achieved via several overlapping mechanisms. HA and NA, that accumulate on one side of the plasma membrane, as well as matrix protein 1 (M1) and M2 have all been discussed to play an important role in producing membrane curvature for budding (Elleman and Barclay, 2004; Chen et al., 2007; Lai et al., 2010; Rossman et al., 2010; Dou et al., 2018; Motsa and Stahelin, 2021; Bao et al., 2022). In general, the shape of lipid bilayer membranes can also be modified by the incorporation of cone-shaped lipids into one side of the bilayer, that are produced by cellular phospholipase A_2_ (PLA_2_) enzymes (Jarsch et al., 2016).

The ester-type local anesthetic procaine was first synthesized in 1904 and is one of the oldest local anesthetics (Tobe et al., 2018). Its classical mechanism of action is the inhibition of voltage-gated sodium channels on nerve cells, which produces anesthesia. Since procaine’s discovery it has been investigated for a number of different purposes and has been found to have several additional effects besides local anesthesia, such as DNA demethylating activity (Villar-Garea et al., 2003), anti-bacterial effects in high concentrations (Schmidt and Rosenkranz, 1970) and anti-inflammatory effects (Doran and Yi, 2007). The activity of some specific enzymes such as PLA_2_ and trypsin is affected by local anesthetics in varying concentrations (Waite and Sisson, 1972; Hendrickson and van Dam-Mieras, 1976; Gayda et al., 1979). Local anesthetics also interact with lipid membranes, which has been suspected as an underlying mechanism of many of their different effects (Tsuchiya and Mizogami, 2013; Krogman et al., 2023). Procaine has interestingly been seen to have antiviral effects on a range of different viruses such as West Nile virus (WNV), herpes simplex virus (HSV), Junin virus and vesicular stomatitis virus (VSV) (Fuchs and Levanon, 1978; Bussereau and Genty, 1980; Castilla et al., 1994). Although it was determined to affect the entry phase of several, but not all of these viruses, overall little is known about the mechanism behind procaine’s antiviral effect. In addition, we recently published results demonstrating an inhibitory effect of procaine derivatives on severe acute respiratory syndrome coronavirus 2 (SARS-CoV-2) replication (Häring et al., 2023).

In the present study we used two derivatives of procaine, ProcCluster^®^ (PC) and procaine hydrochloride (PHCl), and investigated their antiviral activity against IAV *in vitro* using three different cell lines.

## 2 Methods

### 2.1 Cell culture and viruses

A549 cells (ATCC Cat. No. CCL-185, human epithelial lung cancer cell line) were cultivated in DMEM (Sigma-Aldrich, United States) with 10% fetal calf serum (FCS; Anprotec, Germany). MDCK cells (ATCC Cat. No. CCL-34, Madin-Darby canine kidney cell line) and Calu-3 cells (ATCC Cat. No. HTB-55, human lung-adenocarcinoma cell line) were cultivated in MEM (Anprotec, Germany) supplemented with 10% FCS. The virus strains used in this work are influenza virus A/Puerto Rico/8/34 (PR8; H1N1), A/Wisconsin/67/05 (Wis; H3N2), A/Jena/5258/09 (Jena09; H1N1pdm), A/NRW/172/09 (NRW172; H1N1pdm) and A/NRW/173/09 (NRW173; H1N1pdm).

### 2.2 Chemical inhibitors

ProcCluster^®^ (supplied by inflamed pharma GmbH, Germany), PHCl (Chongqing Soutwest No.2 Pharmaceutical Factory, China) and oseltamivir phosphate (Sigma Aldrich, United States) were diluted in ddH_2_O and stored frozen at −20°C. Bromoenol lacton (Merck, Germany) and pyrrophenone (MedChemExpress, United States) were diluted in DMSO and stored at −20°C.

### 2.3 Cell toxicity assays

To determine lactate-dehydrogenase (LDH) release A549 cells (300,000 cells/well), Calu-3 cells (500,000 cells/well) or MDCK cells (350,000 cells/well) were seeded in 12-well plates 1 day (A549 and MDCK) or 2 days (Calu-3) prior to use and were then incubated with PC, PHCl or H_2_O at the indicated concentrations or left untreated. As control lysis buffer supplied by the CyQUANT^™^ LDH Cytotoxicity Assay kit (Thermo Fisher Scientific, United States) was added to the medium of untreated cells for 15 min at 37°C after 24 h. Supernatants were collected 24 h post H_2_O, PC or PHCl-treatment and LDH release was determined using the CyQUANT^™^ LDH Cytotoxicity Assay kit according to the manufacturer’s instructions. Absorption was measured at 490 nm and 680 nm using a FLUOstar Omega plate reader (BMG Labtech, Germany). Absorption at 680 nm was subtracted as background from that at 490 nm.

Light microscopy pictures were taken using an Axio Vert.A1 light microscope (Zeiss, Germany) prior to taking supernatants for the LDH assay.

MTT (3-(4,5-dimethylthiazol-2-yl)-2,5-diphenyltetrazolium bromide) assays were carried out by seeding A549 cells (20,000 cells/well), MDCK cells (20,000 cells/well) or Calu-3 cells (100,000 cells/well) into 96-well plates 24 h prior to use. The cells were treated with the indicated concentrations of PC or PHCl in 100 μL DMEM (A549) or MEM (MDCK, Calu-3) supplemented with 10% FCS for 24 h. At the end of the incubation period 25 μL of 5 mg mL^−1^ MTT were added without removing the medium and the cells were incubated for a further 2 h. The medium was removed and the cells were lysed in 50 μL DMSO. Absorption was measured at 570 nm using a FLUOstar Omega plate reader (BMG Labtech, Germany).

### 2.4 Viral infection and plaque assay

A549, MDCK or Calu-3 cells were seeded 24 h (A549, MDCK) or 48 h (Calu-3) prior to viral infection. A549 cells were seeded with 300,000 cells/well in 12-well plates or 600,000 cells/well in 6-well plates. MDCK cells were seeded with 400,000 cells/well in 12-well or 800,000 cells/well in 6-well plates. Calu-3 cells were seeded at 500,000 cells/well in 12-well plates or 1,000,000 cells/well in 6-well plates. Cells were washed with phosphate buffered saline (PBS) once prior to infection and then incubated with the indicated multiplicity of infection (MOI) of IAV in PBS_Inf_ [PBS supplemented with 0.2% BSA (Carl Roth, Germany), 1 mM MgCl_2_ and 0.9 mM CaCl_2_] for 30 min at 37°C and 5% CO_2_. After the initial infection cells were incubated in DMEM_Inf_ (A549) or MEM_Inf_ (MDCK, Calu-3) until the desired time post infection. DMEM_Inf_ and MEM_Inf_ consist of DMEM or MEM respectively, supplemented with 0.2% BSA, 1 mM MgCl_2_, 0.9 mM CaCl_2_ and 0.17 μg mL^−1^ TPCK trypsin (Sigma-Aldrich, United States).

Virus titers were determined by standard plaque assays. MDCK cells were seeded in 6-well plates (2,000,000 cells/well) to form a 90% confluent layer and infected with serial dilutions of the sample in PBS_Inf_ supplemented with penicillin/streptomycin (pen/strep) (100 U mL^−1^pen/0.1 mg mL^−1^ strep) for 30 min at 37°C and 5% CO_2_. The PBS_Inf_ was aspirated and replaced by MEM supplemented with 0.2% BSA, 0.01% DEAE Dextran (Pharmacia Biotech, Germany), 0.2% NaHCO_3_ (Biozym Scientific, Germany), 100 U mL^−1^/0.1 mg mL^−1^ pen/strep, 0.25 μg mL^−1^ TPCK trypsin and 0.9% agar (Oxoid, Germany) and the cells were incubated at 37°C and 5% CO_2_ for 3 days. Plaques were visualized using neutral red or crystal violet staining.

### 2.5 Western blot

Triton lysis buffer (20 mM Tris-HCl, pH 7.4; 137 mM NaCl; 10% Glycerol; 1% Triton X-100; 2 mM EDTA; 50 mM sodium glycerophosphate, 20 mM sodium pyrophosphate; 0.2 mM Pefabloc^®^, 5 μg mL^−1^ aprotinin; 5 μg mL^−1^ leupeptin; 1 mM sodium vanadate and 5 mM benzamidine) was used to lyse cells for 30 min while kept at 4°C. Cell lysates were cleared by centrifugation (10 min, 14,000 rpm, 4°C) and protein content was measured using Protein Assay Dye Reagent Concentrate (BioRad, United States) and samples were diluted appropriately for equal protein amounts. Samples were supplemented with a 5× Laemmli buffer (10% SDS, 50% glycerol, 25% 2-mercaptoethanol, 0.1% bromophenol blue, 312 mM Tris pH 6.8) in a 1:5 ratio and heated to 95°C for 10 min. Samples were subjected to SDS-PAGE and blotting. Proteins were detected using the primary antibodies listed in Table 1 and WesternSure^®^ HRP goat anti-mouse or goat anti-rabbit IgG (LI-CORE Biosciences, United States) as secondary antibody. Quantification normalized to the α-tubulin loading control was done using Fiji (Image J.JS v0.5.7; https://ij.imjoy.io).

**Table 1:** Antibodies for western blot analysis.

Target	Manufacturer
IAV PB1	GeneTex (GTX125923)
IAV HA	GeneTex (GTX127357)
IAV M1	BioRad (MCA401)
α-tubulin	Cell signaling (2144)

### 2.6 Virus-endosome fusion assay

To investigate the fusion of the viral envelope with the endosome, IAV PR8 stock solution [1.13 × 10^8^ plaque forming units (PFU) mL^−1^] was incubated with 0.2 μM 3,3′-dioctadecyl-5,5′-di(4-sulfophenyl)oxacarbocyanine (SP-DiOC18) (Abcam, United Kingdom) and 0.4 μM octadecyl rhodamine B chloride (R18) (Sigma Aldrich, United States) for 1 h at room temperature. The solution was filtered using a sterile 0.45 μm filter. A549 cells were pre-incubated with 2 μg mL^−1^ U18666A (U18) (Sigma Aldrich, United States) for 18 h prior to infection as positive control. Others were left untreated. Cells were infected with the dye-treated virus particles with an MOI of 10 for 30 min on ice followed by 30 min incubation at 37°C. Mock-infected cells were incubated with a solution of R18 and SP-DiOC18 in PBS that was treated in an identical manner to the virus solution. Cells were thereafter incubated with 2.5 mM PC or PHCl for the indicated times. Cells were detached using accutase (Biozym Scientific, Germany), washed once with PBS and fixed with 2% formaldehyde for 30 min at room temperature. Cells were washed, resuspended in PBS and the percentage of SP-DiOC18 positive cells (determined by comparison with the mock-infected control) out of 10,000 cells was measured by flow cytometry using a BD FACSLyric^™^ flow cytometer (BD Biosciences, United States).

### 2.7 Quantitative real time PCR

To isolate RNA from A549, MDCK or Calu-3 cells we used the RNeasy Mini Kit (Qiagen, Germany) according to the manufacturer’s instruction. The QuantiTect Reverse Transcription Kit (Qiagen, Germany) was used for copy DNA (cDNA) synthesis from 400 ng of total RNA from cells. For quantitative real time PCR (qRT-PCR) the QuantiNova SYBR Green PCR Kit (Qiagen, Germany) was used. Cycle conditions were set as follows: 95°C for 2 min, followed by 40 cycles of 95°C for 5 s and 60°C for 10 s. The PCR cycle concluded with a stepwise temperature-increase from 60°C to 95°C (1°C every 5 s). All primers used are listed in Table 2 (fw: forward, rv: reverse). The mRNA expression relative to glyceraldehyde 3-phosphate dehydrogenase (GAPDH) mRNA expression was calculated using the 2^−ΔΔCT^-method (Livak and Schmittgen, 2001).

**Table 2:** Primers.

Name	Sequence
IAV Segment7 fw	5′-GAC CAA TCC TGT CAC CTC TGA C-3′
IAV Segment7 rv	5′-AGG GCA TTT TGG ACA AAG CGT CTA-3′
Human GAPDH fw	5′-CTC TGC TCC TCC TGT TCG AC-3′
Human GAPDH rv	5′-CAA TAC GAC CAA ATC CGT TGA C-3′
Human IFNβ fw	5′-ATG ACC AAC AAG TGT CTC CTC C-3′
Human IFNβ rv	5′-GGA ATC CAA GCA AGT TGT AGC TC-3′
Human MxA fw	5′-GAA GGG CAA CTC CTG ACA G-3′
Human MxA rv	5′-GTT TCC GAA GTG GAC ATC GCA-3′
Human IP10 fw	5′-CCA GAA TCG AAG GCC ATC AA-3′
Human IP10 rv	5′-TTT CCT TGC TAA CTG CTT TCA G-3′
Human IFNλ1 fw	5′-CGC CTT GGA AGA GTC ACT CA-3′
Human IFNλ1 rv	5′-GAA GCC TCA GGT CCC AAT TC-3′
Human IFNλ2/3 fw	5′-AGT TCC GGG CCT GTA TCC AG-3′
Human IFNλ2/3 rv	5′-GAG CCG GTA CAG CCA ATG GT-3′
Human HSPA5 fw	5′-CTG TCC AGG CTG GTG TGC TCT-3′
Human HSPA5 rv	5′-CTT GGT AGG CAC CAC TGT GTT C-3′
Human HSP90B1 fw	5′-GGA GAG TCG TGA AGC AGT TGA G-3′
Human HSP90B1 rv	5′-CCA CCA AAG CAC ACG GAG ATT C-3′
Human DDIT3 fw	5′-GGT ATG AGG ACC TGC AAG AGG T-3′
Human DDIT3 rv	5′-CTT GTG ACC TCT GCT GGT TCT G-3′

### 2.8 Hemagglutination and neuraminidase activity assay

Human erythrocyte concentrate was purchased from the Institute of Transfusion Medicine of the University Hospital Jena. Human erythrocyte concentrate (blood type 0) was washed three times with PBS and diluted to an appropriate concentration (OD_600_ = 1.1–1.2 after 1:10 dilution) in PBS. Optimal dilution of the IAV stock was determined by preliminary experiments. Erythrocytes were then incubated with IAV PR8 and a dilution series of PC or PHCl in PBS in V-bottom 96-well plates at 4°C for 2 h, after which wells showing hemagglutination of the erythrocytes were documented. The plate was then moved to 37°C over night after which the state of each well was again noted. The hemagglutination pattern of PC or PHCl-treated samples was evaluated compared to untreated erythrocytes.

### 2.9 Phospholipase A_2_ assay

A549 or MDCK cells were collected in PBS from cell culture plates using a cell scraper and were centrifuged at 4°C and 4,500 rpm for 10 min. Supernatants were discarded and replaced by ddH_2_O. Cells were resuspended by vortexing and kept on ice for 30 min, after which equal amounts of 2x reaction buffer (EnzChek^™^ Phospholipase A_2_ Assay Kit, Thermo Fisher Scientific, United States) were added. Samples were centrifuged at 4°C and 4,500 rpm for 10 min. PLA_2_ activity of these lysates with and without the addition of different concentrations of PC or PHCl was analyzed using the EnzChek^™^ Phospholipase A_2_ Assay Kit (Thermo Fisher Scientific, United States) according to the manufacturer’s instructions. Fluorescence was measured using a FLUOstar Omega plate reader (BMG Labtech, Germany) (Excitation: 485 nm; Emission: 520 nm).

### 2.10 Infrared spectroscopy measurements

Infrared spectroscopy was conducted using attenuated total reflection (ATR) spectroscopy on a Nicolet^™^ Avatar 380 FT-IR spectrometer.

### 2.11 Solubility measurements in water and octanol

PHCl and PC were added to water or octanol in a 10 mL volumetric flask to a theoretical concentration of 0.046 mM. After 2 h, 250 μL of the solution were added to a new flask with 10 mL of the respective solvent (water or octanol) and a UV/VIS spectrum was obtained.

## 3 Results

### 3.1 Properties of ProcCluster^®^ and procaine hydrochloride

PHCl (1) and PC (2) are drugs based on the active component procaine (3) (Table 3). PC is the hydrogen carbonate of procaine while PHCl, as the name suggests, is the hydrochloride of procaine. PC, or procainium hydrogencarbonate, is stabilized by the mineral salt sodium chloride. Structural differences are visualized using infrared spectroscopy in Supplementary Figure S1. PC, PHCl and procaine differ in their melting temperature (or in the case of PC decomposition temperature) as well as in their pH value (1% solution), with procaine having the highest (pH 10.4) and PHCl the lowest (pH 6.1) (Table 3). Measurements of the solubility of these substances in octanol demonstrate a much higher solubility of PC than procaine hydrochloride (Supplementary Figure S2), which would indicate improved membrane penetration of PC compared to PHCl. The lipophilic procaine is poorly soluble in water, unlike the hydrophilic PHCl and the amphiphilic PC (Table 3).

**Table 3:** Properties of procaine hydrochloride, ProcCluster^®^ and procaine.

Name	Procaine hydrochloride	ProcCluster^®^	Procaine
Structure	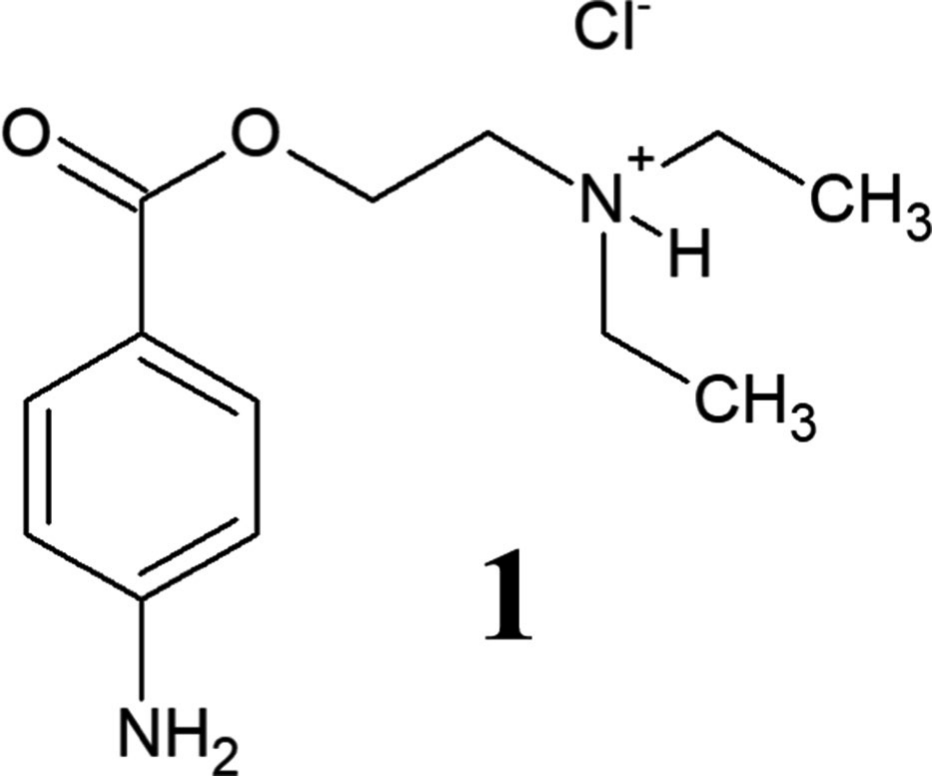	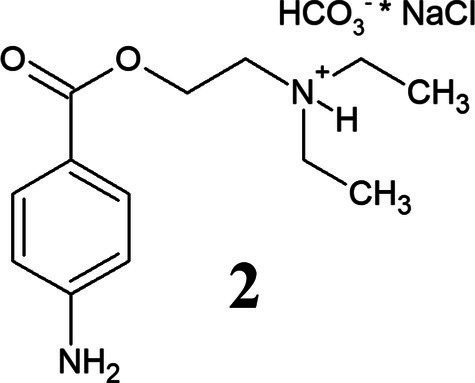	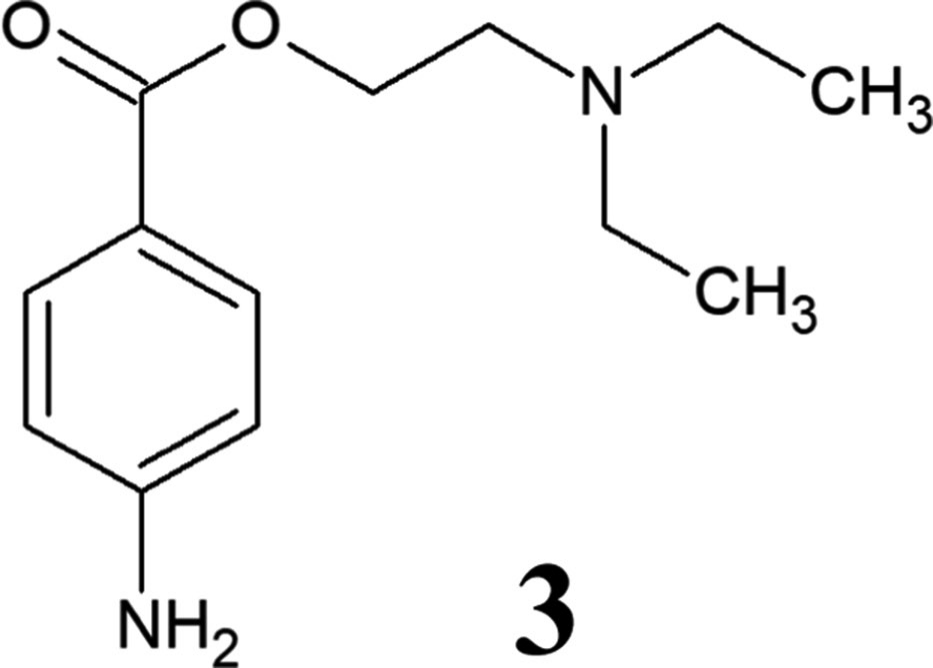
Solubility	Good in water	Good in water	Organic solvents
Melting point	157–158°C	>71°C (decomposition temperature)	60–61°C
pH (1% solution)	6.1	7.7	10.4
Membrane permeation	Low	Good	Good

### 3.2 ProcCluster^®^ and procaine hydrochloride treatment cause a reduction in IAV titers

**Figure 1:**
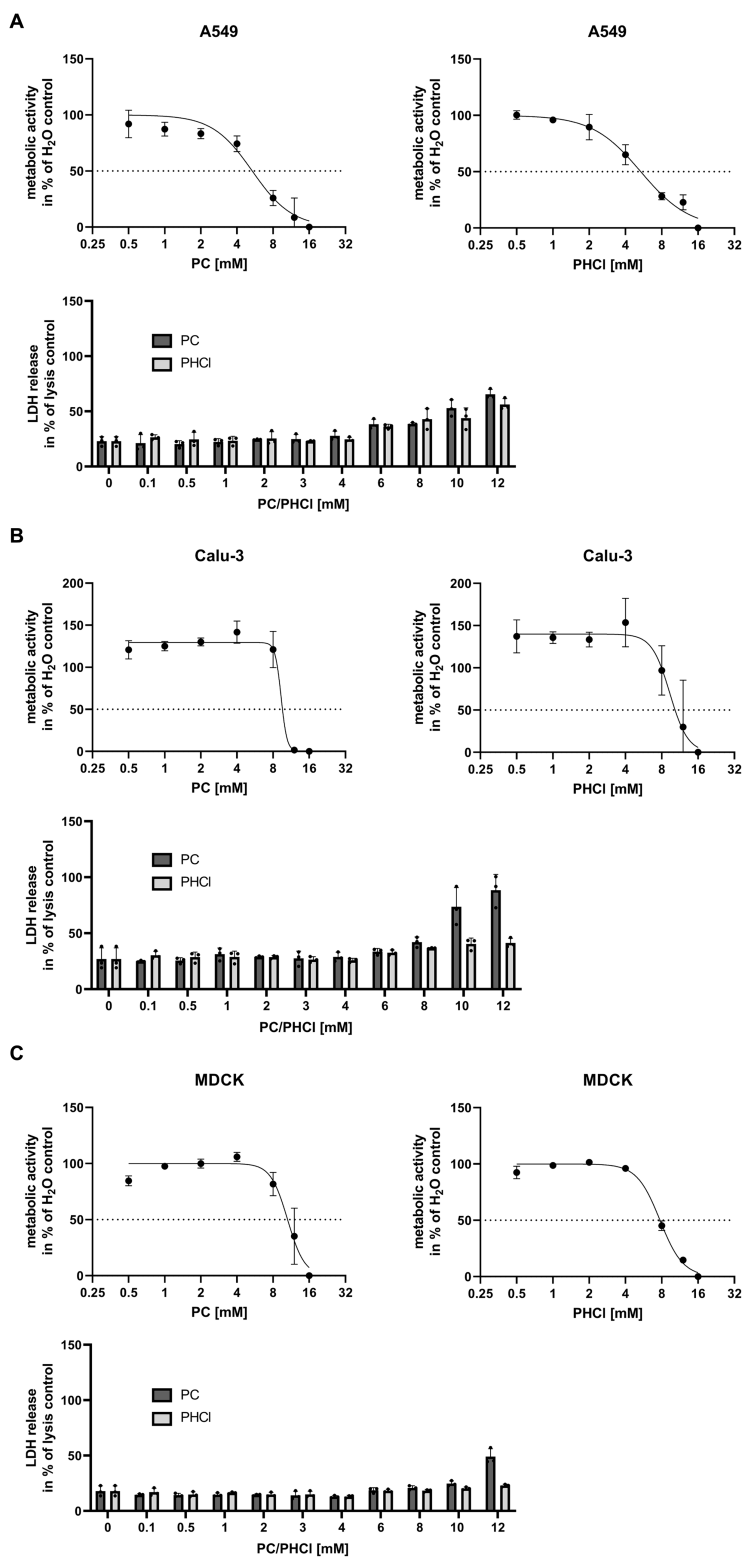
Metabolic activity and LDH release after 24 h treatment with PC and PHCl. **(A)** A549, **(B)** Calu-3 and **(C)** MDCK cells were treated with the indicated concentrations of solvent control (H_2_O), PC or PHCl for 24 h and analyzed by MTT- (upper panels) or LDH-assay (lower panels). For MTT-assay the metabolic activity was normalized to the H_2_O-treated sample (100%) and the 16 mM PC or PHCl-treated sample (0%). The mean ± SD of three independent experiments is depicted. GraphPad Prism (v.9.3.1) was used to perform a curve fit by non-linear regression. For LDH-assay the untreated cells were lysed 24 h post treatment as maximum LDH release control. The supernatants of all samples were used to determine LDH release. The data represent the mean + SD of three independent experiments as percentage of maximum LDH release.

Cellular metabolic activity was determined by MTT assay in Calu-3, A549 and MDCK cells in order to identify tolerable concentrations of PC and PHCl *in vitro* (Figures 1A–C, upper panels). In A549 cells PC and PHCl caused a 50% reduction in metabolic activity at 5.36 mM [95% confidence interval (CI) 4.45 mM–6.32 mM] and 5.35 mM (CI 4.76 mM–6.00 mM), respectively. A reduction in metabolic activity in Calu-3 and MDCK cells was only observed in this assay upon treatment with high concentrations (around 10 mM) of PC or PHCl, when cells already showed strong morphological changes visible in light microscopy. We therefore did not calculate an inhibitory concentration in these cells. To further investigate potential cytotoxic effects of PC and PHCl, we performed LDH release measurements (Figures 1A–C, lower panels) and documented cell morphology using light microscopy pictures (Supplementary Figure S3). LDH release indicates apoptotic or necrotic damage to the cells. LDH release after 24 h treatment with PC, PHCl or H_2_O control was compared to the maximum LDH release caused by addition of lysis buffer to the cells after 24 h without inhibitor treatment. Again A549 cells showed an increase in LDH release in the presence of 6 mM and more of PC or PHCl while lower concentrations caused the same amount of LDH release as the solvent control. Calu-3 and MDCK cells however did not show an increase in LDH release until all cells were visibly detached (10–12 mM PC). The cell morphology of Calu-3 and A549 cells appears unchanged after treatment with up to 4 mM PC or PHCl. Treatment with 6 mM PC or PHCl causes the formation of large intracellular vacuole structures that increase in number with increasing treatment concentrations. The same intracellular vacuoles appeared in MDCK cells after treatment with a concentration of 3 mM or more of PC or PHCl (Supplementary Figure S3). Although these changes did not correlate directly with measurable changes in metabolic activity or LDH release, we used a maximum concentration of 2.5 mM for further experiments in all three cell lines.

**Figure 2:**
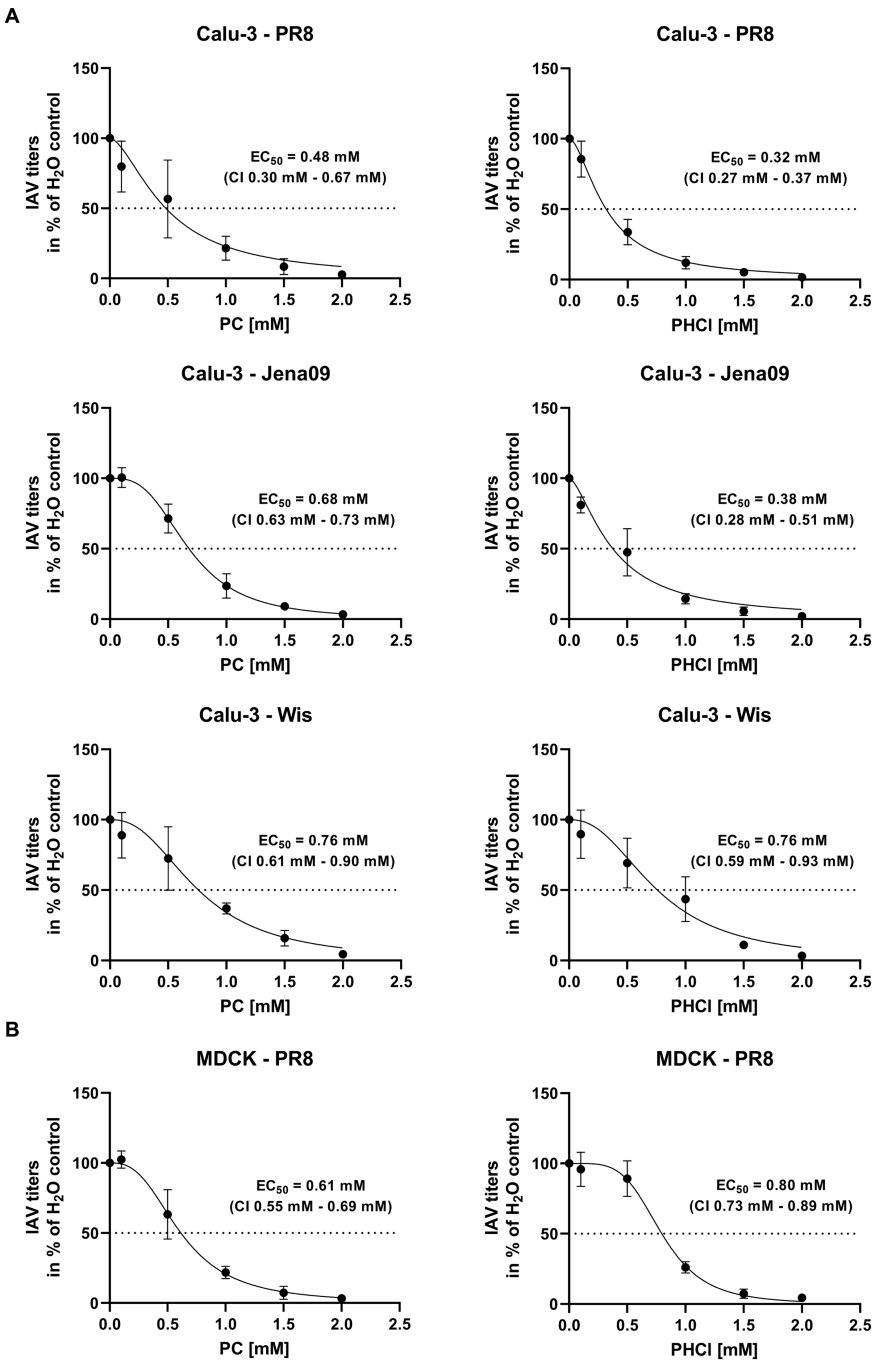
PC and PHCl treatment cause an inhibition of IAV replication *in vitro*. **(A)** Calu-3 cells or **(B)** MDCK cells were infected with either A/Puerto Rico/8/34 (PR8) **(A,B)**, A/Jena/5258/09 (Jena09) **(A)** or A/Wisconsin/67/05 (Wis) **(A)** at an MOI of 0.1 for 30 min. The cells were washed with PBS and incubated with PC or PHCl or solvent control (H_2_O). After 24 h of incubation supernatants were collected and progeny virus titers were determined by standard plaque assay. Virus titers are shown as percentage of the solvent control. The mean ± SD of three independent experiments with two biological replicates is depicted. Effective concentration 50% (EC_50_) values and 95% confidence intervals (CI) were determined by non-linear regression in GraphPad Prism (v.9.3.1).

Multi-cycle infection experiments were used to determine whether PC and PHCl have an inhibitory effect on IAV replication. Calu-3 cells were infected with an MOI of 0.1 of three different IAV isolates; the H1N1 strain A/Puerto Rico/8/34 (PR8), the H1N1pdm isolate A/Jena/5258/09 (Jena09) from the 2009 swine flu pandemic and the H3N2 strain A/Wisconsin/67/05 (Wis) (Figure 2A). After infection the cells were treated with PC or PHCl for 24 h in concentrations from 0.1 to 2 mM. Both substances caused a dose-dependent reduction in virus titers with all three virus strains with effective concentration 50% (EC_50_) values ranging from 0.32 mM to 0.76 mM. The corresponding confidence intervals for the individual EC_50_ values do not offer good evidence that effectiveness differs depending on the virus strain used. PC and PHCl also caused a dose-dependent reduction of virus titers in PR8-infected MDCK cells, with EC_50_ values of 0.61 mM and 0.80 mM, respectively (Figure 2B).

**Figure 3:**
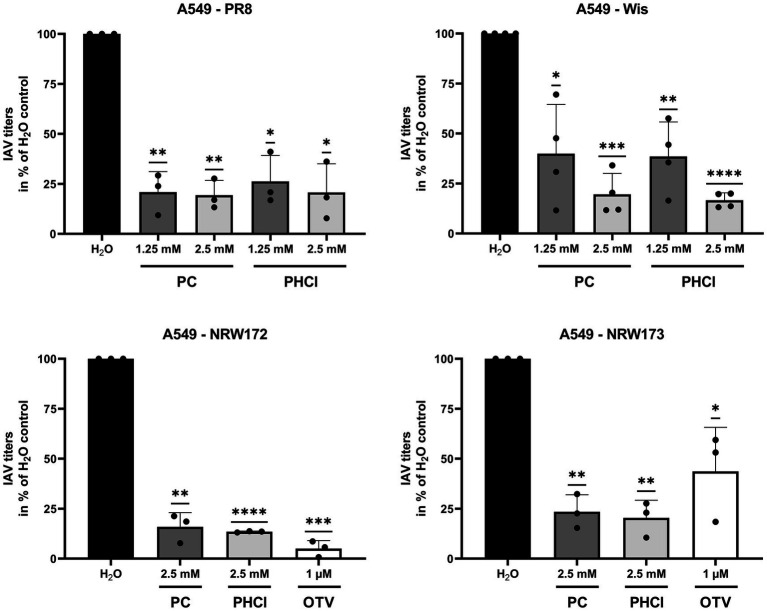
PC and PHCl are effective against oseltamivir-tolerant viruses. A549 cells were treated with the indicated concentrations of PC, PHCl, solvent control (H_2_O) or oseltamivir (OTV) for 30 min and then infected with A/Puerto Rico/8/34 (PR8), A/Wisconsin/67/05 (Wis), A/NRW/172/09 (NRW172) or A/NRW/173/09 (NRW173) as indicated at an MOI of 0.1 (PR8 and Wis) or 0.5 (NRW172 and NRW173) for 30 min. Cells were then incubated with the indicated concentrations of PC, PHCl or OTV until 24 h p.i. Virus titers were determined by standard plaque assay. The virus titer in the solvent control sample was arbitrarily set to 100%. The mean + SD of at least three independent experiments with two biological replicates is depicted. Statistical significance was determined by one-sample *t*-test comparing to 100. ^*^p < 0.05, ^**^p < 0.01, ^***^p < 0.001, and ^****^p < 0.0001.

PC and PHCl also significantly inhibited the replication of IAV PR8 and Wis in A549 cells using concentrations of 1.25 and 2.5 mM (Figure 3, upper panels). Virus titers of IAV PR8 where reduced by around 80% compared to the solvent control at both concentrations. IAV Wis titers with 1.25 and 2.5 mM PC or PHCl treatment were reduced by around 60 and 80%, respectively. Both substances (used at 2.5 mM) were also able to inhibit the replication of A/NRW/172/09 (NRW172; H1N1pdm) and A/NRW/173/09 (NRW173; H1N1pdm) (Figure 3, lower panels). NRW172 and NRW173 are two isolates from the same patient, taken from an early sample and after oseltamivir treatment of the patient, respectively, making the later less oseltamivir sensitive. The effect of 1 μM oseltamivir treatment is noticeably reduced in the later isolate, while the effect of PC and PHCl remains.

### 3.3 PC and PHCl show cell line-dependent differences in their effects on the IAV replication cycle

**Figure 4:**
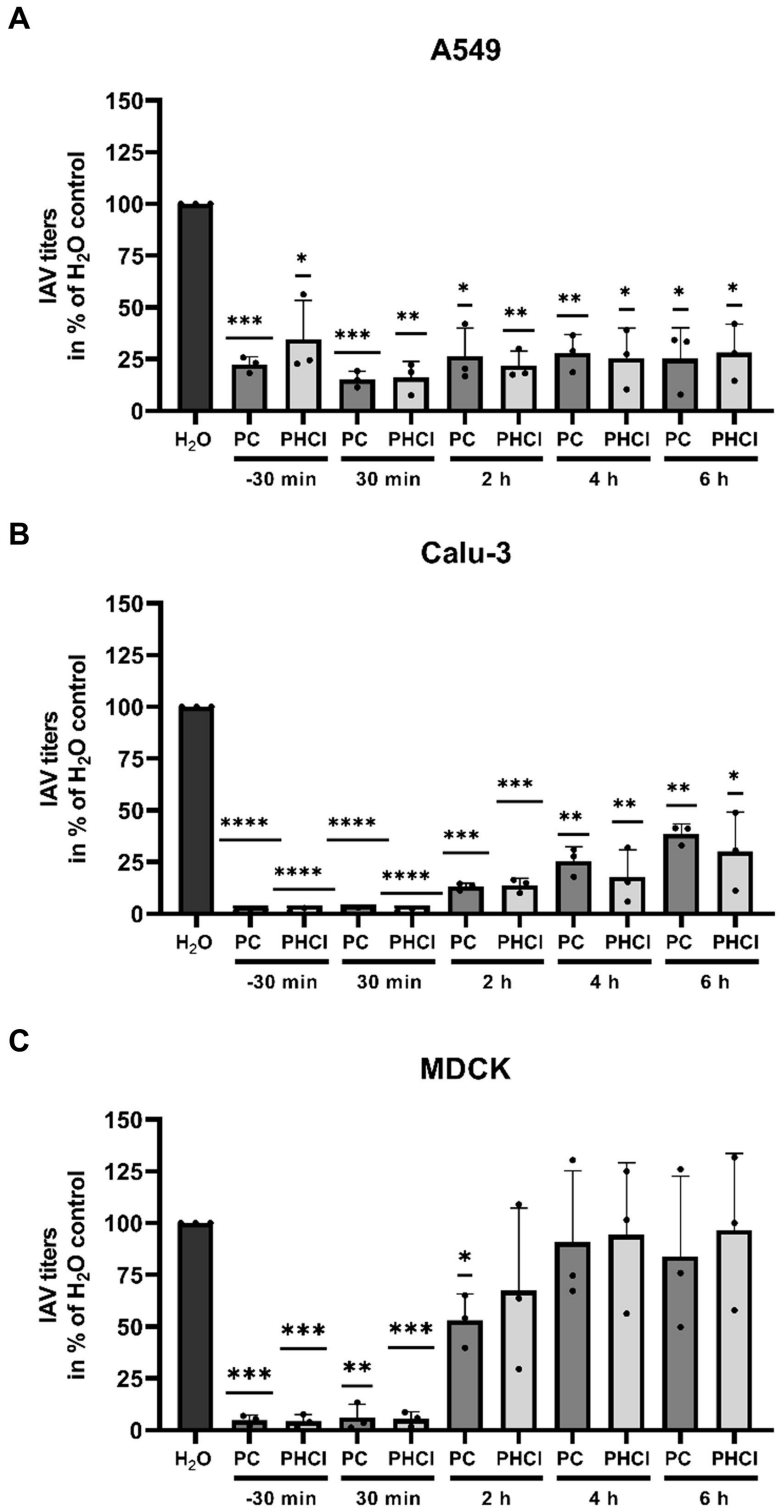
PC and PHCl treatment result in a reduction of progeny virus titers as late as 6 h p.i. in A549 and Calu-3 cells. **(A)** A549, **(B)** Calu-3 and **(C)** MDCK cells were incubated with 2.5 mM PC or PHCl (−30 min) or were left untreated for 30 min prior to infection. The cells were infected with IAV PR8 at an MOI of 1 for 30 min and then again treated with 2.5 mM PC, PHCl or solvent control (H_2_O) either immediately (−30 min, 30 min and H_2_O) or at the indicated times without removing the media. Viral titers were analyzed at 9 h p.i. by standard plaque assay. The solvent control was arbitrarily set to 100%. The mean + SD of three independent experiments with two biological replicates is depicted. Statistical significance was determined by one-sample *t*-test comparing to 100. ^*^p < 0.05, ^**^p < 0.01, ^***^p < 0.001, and ^****^p < 0.0001.

To determine at which stage of the replication cycle procaine treatment limits progeny virus production we performed time of addition experiments in which we added PC or PHCl starting at different times pre and post infection with PR8 (MOI 1) and determined viral titers after 9 h (Figure 4). Interestingly, in A549 cells the reduction in virus titers by around 70% under treatment with 2.5 mM PC or PHCl was consistent throughout the replication cycle even when the substances were added as late as 6 h p.i. (Figure 4A). In Calu-3 cells, the reduction in virus titers was most pronounced when treatment was started directly after infection (over 90% reduction). Treatment at later times became increasingly less effective, but at 6 h p.i. still reduced virus titers by 60% (Figure 4B). The reduction in efficacy at later points in the replication cycle was even more pronounced in MDCK cells. Treatment with PC or PHCl directly after infection again inhibited virus titers by over 90% but had only a minor effect at 2 h p.i. and no effect starting from 4 h p.i. (Figure 4C). PC and PHCl treatment seem to affect virus replication early on in all three cell lines and have an additional effect at a very late stage of the replication cycle in A549 and Calu-3 cells but not in MDCK cells. Pre-incubation of Calu-3 cells for 16 h or 1 h prior to infection without treatment after infection had no effect on virus titers (Supplementary Figure S4).

### 3.4 PC and PHCl treatment cause a delay in IAV RNA and protein synthesis

**Figure 5:**
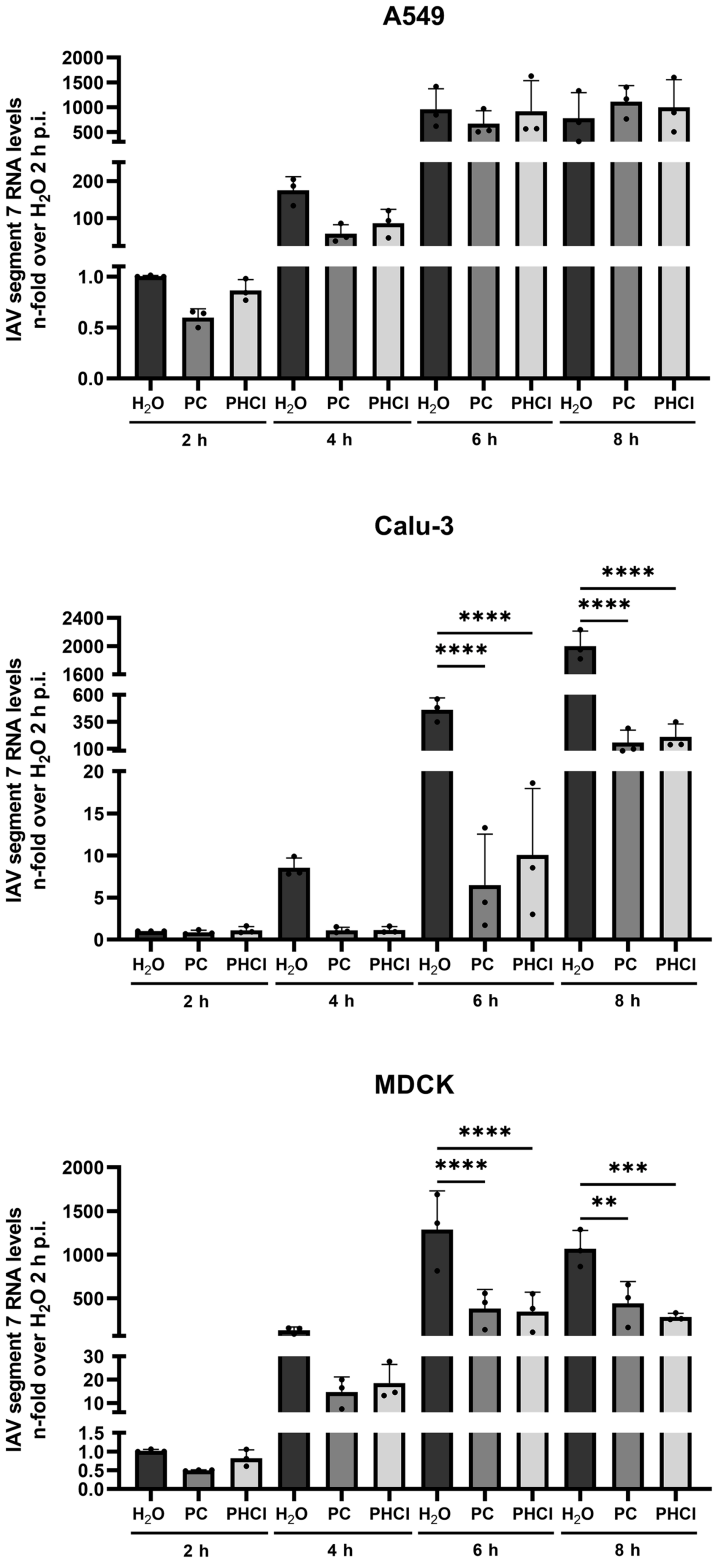
PC and PHCl treatment inhibit IAV RNA synthesis. A549, Calu-3 or MDCK were infected with IAV PR8 (MOI 5) for 30 min and were afterwards treated with 2.5 mM PC or PHCl or solvent control (H_2_O). The cells were washed and lysed for RNA extraction at 2, 4, 6 and 8 h p.i. A qRT-PCR was conducted to determine the relative amount of IAV RNA segment 7 using GAPDH levels for normalization. RNA levels of the solvent control (H_2_O) samples at 2 h p.i. were arbitrarily set to 1. The mean + SD of three independent experiments is depicted. Statistical significance was determined by one-way ANOVA with Bonferroni’s multiple comparisons test. ^**^p < 0.01, ^***^p < 0.001, and ^****^p < 0.0001.

To further determine how procaine treatment interferes with IAV infection, the levels of IAV RNA for segment 7 and viral protein synthesis were investigated at different times during the first replication cycle. Cells were infected with IAV PR8 (MOI 5) and treated with 2.5 mM PC, PHCl or solvent control (H_2_O) starting 30 min p.i. Cells were then lysed for RNA at 2, 4, 6 and 8 h p.i. and IAV RNA (segment 7) content was analyzed by qRT-PCR (Figure 5). In A549 cells the RNA amounts of IAV segment 7 were slightly reduced in the PC-treated samples at 2 h p.i. while those from infected cells treated with PHCl were not affected. However, at 4 h p.i., both the PC- and PHCl-treated infected A549 cells showed reduced amounts (by around 50%) of IAV RNA in comparison to control. This reduction was still present but less pronounced at 6 h p.i. and completely absent at 8 h p.i. In Calu-3 and MDCK cells on the other hand a strong reduction in the amount of IAV RNA can be seen from 4 h p.i. that remains for the 6 and 8 h time points. As would be expected a strong increase in the amount of IAV RNA can be seen over time, reaching at peak around 1,000 to 2,000-times the amount at 2 h p.i. Peak amounts were notably reached at 6 h p.i. in A549 and MDCK cells while IAV RNA amounts tripled from 6 to 8 h p.i. in Calu-3 cells.

**Figure 6:**
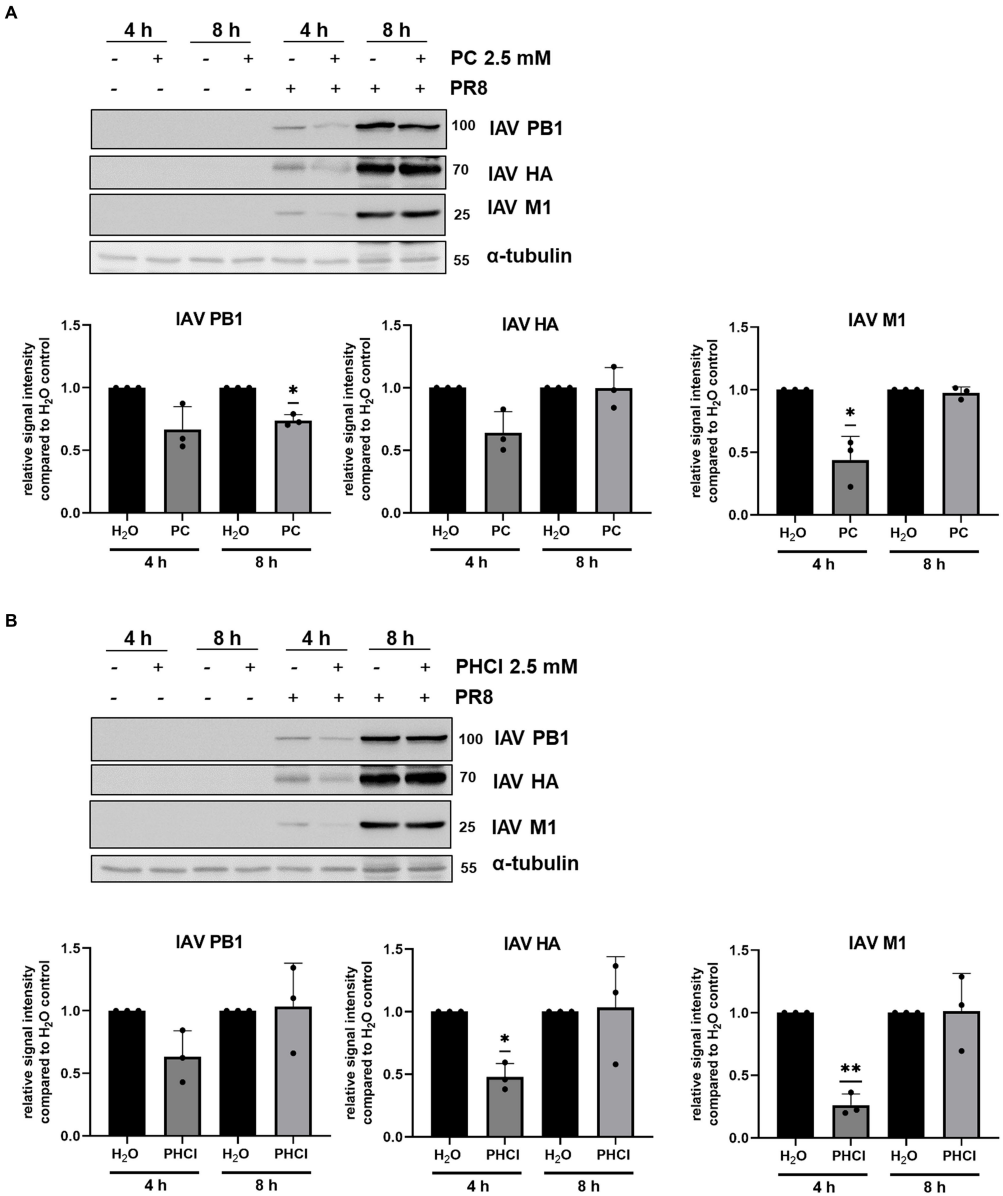
PC and PHCl treatment transiently affect IAV protein synthesis. A549 cells were infected with 3 MOI of IAV PR8 for 30 min and were afterwards treated with 2.5 mM PC **(A)**, PHCl **(B)** or solvent control (H_2_O) **(A,B)**. Cells were washed and lysed for western blot analysis at 4 or 8 h p.i. IAV proteins PB1, HA and M1 were detected using specific antibodies. Cellular α-tubulin was detected as loading control and used to normalize expression levels. The relative protein amount was quantified using Fiji (Image J). Signal intensity in the solvent-treated samples was arbitrarily set to 1. All graphs show the mean + SD of three independent experiments. Blots show one representative example of three independent experiments. Statistical significance was determined by one-sample *t*-test comparing to a hypothetical mean of 1. ^*^p < 0.05 and ^**^p < 0.01.

Because A549 cells showed this more unusual pattern of IAV RNA synthesis under treatment we further investigated the expression of viral proteins in this cell line using western blotting. The expression of the viral proteins polymerase basic protein 1 (PB1), M1 and HA were in line with the qRT-PCR results. Both PC and PHCl treatment provoked a visible reduction in viral protein expression in comparison to solvent treatment at 4 h p.i., but not at 8 h p.i (Figure 6). Thus, these results indicate that in A549 cells the synthesis of both IAV RNA and protein largely recovers from the initial delay caused by procaine treatment.

**Figure 7:**
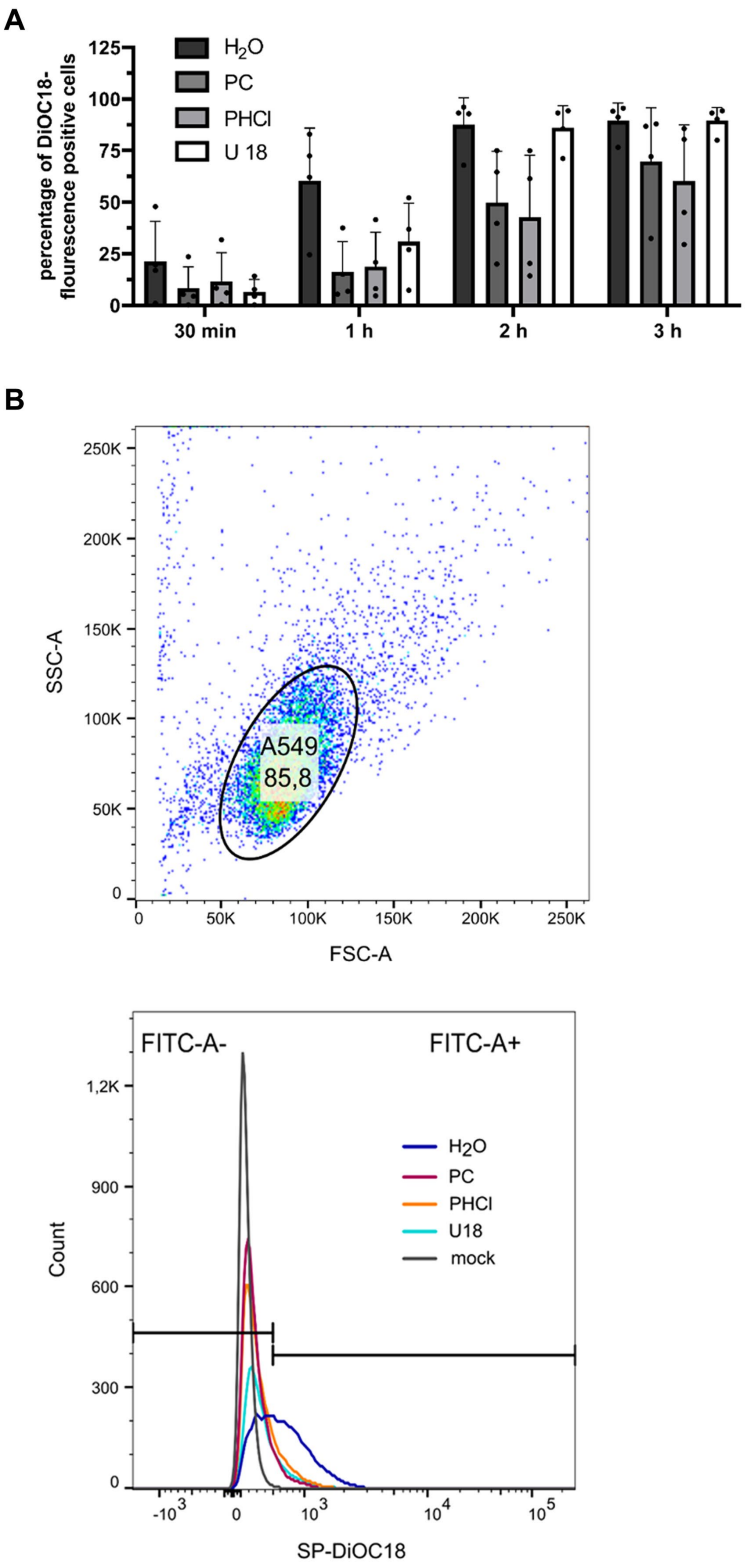
PC or PHCl treatment results in a delay of IAV RNA release from the endosome. **(A)** A549 cells were incubated with 2 μg mL^−1^ U 18 for 18 h or left untreated. IAV PR8 stock-solution was treated with 200 nM SP-DiOC18 and 400 nM R18 for 1 h. The cells were then infected with this IAV PR8 (MOI 10) for 30 min on ice followed by 30 min at 37°C. Afterwards the cells were treated with 2.5 mM of PC or PHCl or solvent control (H_2_O) for the indicated times. The cells were detached using accutase and the percentage of SP-DiOC18 fluorescence positive cells was assessed using a flow cytometer. Positive cells were those showing fluorescence above the values of mock-infected control cells. The graph shows the mean + SD of four independent experiments. Statistical significance was tested using two-way ANOVA with multiple comparisons. **(B, top panel)** Representative depiction of flow cytometry gating for A549 single cell population at 1 h p.i. (H_2_O-treated sample). **(B, bottom panel)** Representative depiction of cutoff (black horizontal lines) used to distinguish SP-DiOC18 fluorescence positive and negative cells by comparison to mock-infected sample. Colored curves show cell number by fluorescence intensity.

Since PC and PHCl treatment was started only 30 min p.i. in the above experiment, attachment and uptake of the viral particle cannot have been affected in this setup. However, fusion of the viral membrane with the endosome and release of the viral ribonucleoproteins could still be altered. Procaine has previously been described to potentially increase the pH of endosomes which would impair IAV release (Bussereau and Genty, 1980). Flow cytometry analysis using dye loaded virus particles can be used to follow virus release from the endosome (Sakai et al., 2006). We loaded IAV PR8 stock solution with the fluorescent dyes 3,3′-dioctadecyl-5,5′-di(4-sulfophenyl)oxacarbocyanine (SP-DiOC18) and octadecyl rhodamine B chloride (R18) in high concentrations. The fluorescent dyes are integrated into the virus particle membrane, where their fluorescence is quenched due to the close proximity of the two dyes. We then infected A549 cells with these virus particles (MOI 10). When the viral envelope fuses with the endosomal membrane, releasing the content of the viral particle from the endosome, the dyes diffuse across the endosomal membrane, which reduces their proximity and strongly increases the fluorescence of SP-DiOC18. This allows the measurement of virus-endosome fusion by detecting SP-DiOC18 fluorescence by flow cytometry. A549 cells were infected for 30 min and then treated with PC or PHCl for 30 min to 3 h. We also treated cells with U18666A (U18) for 18 h prior to infection but not after infection. U18 causes accumulation of cholesterol in the endosomal membrane, which inhibits virus-endosome fusion (Kuhnl et al., 2018). After 30 min around 20% of the solvent-treated control cells were positive for SP-DiOC18 fluorescence, while only around 10% of the PC and PHCl-treated cells were positive (Figure 7). The percentage of positive control cells then increased steeply to around 60% at 1 h and reached 90% after 2 h. The amount of positive PC and PHCl-treated cells only reached about 15% after 1 h and then increased to approximately 40% at 2 h. PC and PHCl-treated cells reached levels similar to those of control cells after 3 h. U18 pre-treatment caused a reduction in the number of positive cells in the first hour but showed the same level as the control cells at 2 h and 3 h. Due to a strong variability in the overall percentage of SP-DiOC18 fluorescence positive cells between the different experimental replicates none of the effects described in this experiment were statistically significant. However, the described reduction of virus endosome fusion with PC or PHCl treatment could be seen in every experimental replicate. PC and PHCl treatment seem to delay the release of viral material from the endosome, which would explain the delay in viral RNA and protein synthesis observed in A549 cells. The fact that both RNA and protein synthesis reach levels near those of the control cells indicates however that this is not the main mechanism responsible for the reduction in virus titers observed in A549 cells.

**Figure 8:**
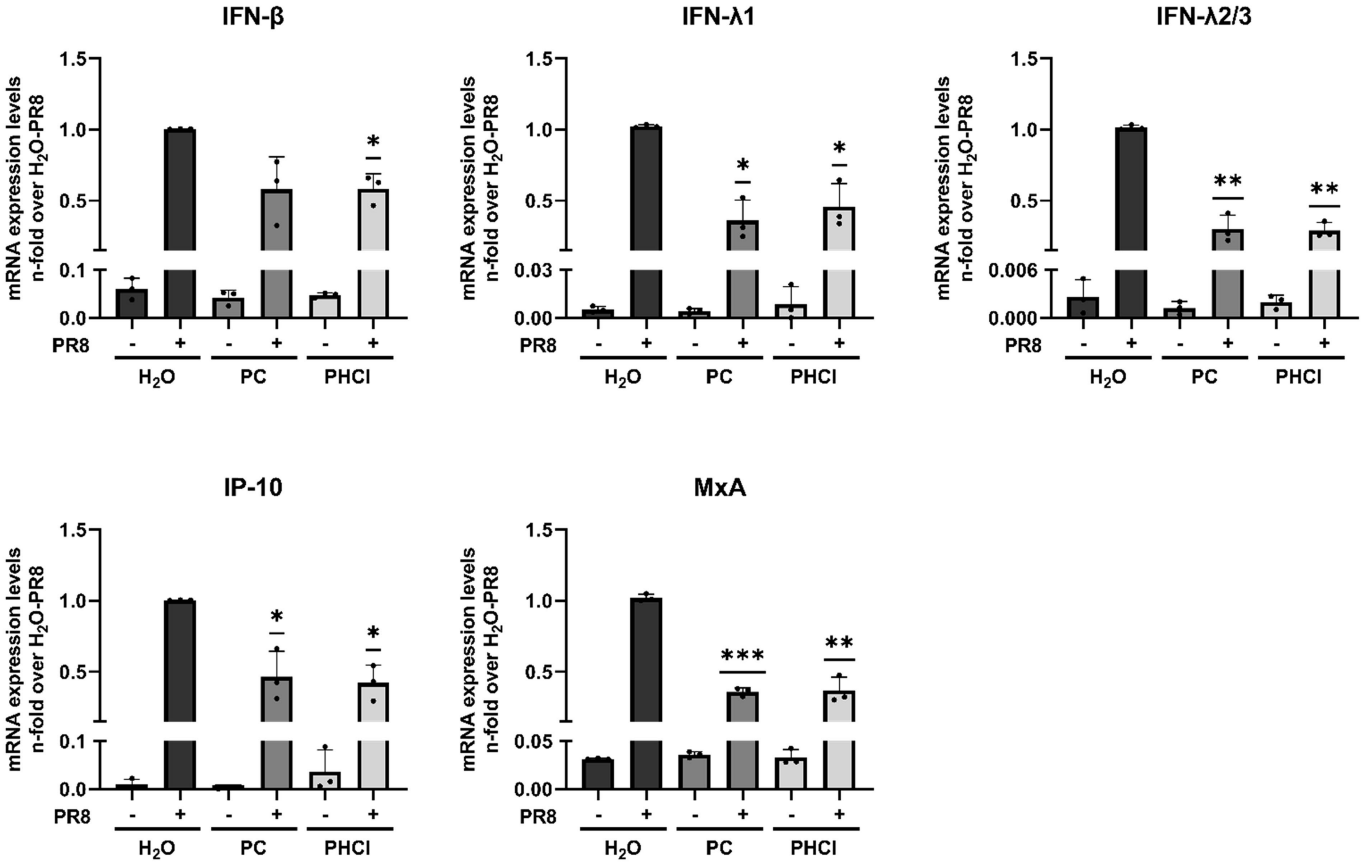
PC and PHCl treatment cause a reduction in the anti-IAV IFN-response at 6 h p.i. A549 cells were infected with IAV PR8 (MOI 5) for 30 min and were afterwards treated with the indicated concentrations of PC or PHCl or solvent control (H_2_O). RNA lysates were performed at 6 h p.i. A qRT-PCR was conducted to determine the relative amount of mRNA for the indicated genes using GAPDH levels for normalization. The mRNA expression of the untreated IAV-infected samples was arbitrarily set to 1. The mean + SD of three independent experiments with two biological replicates is depicted. Statistical significance was determined by one-sample *t*-test comparing to a hypothetical mean of 1. ^*^p < 0.05, ^**^p < 0.01, and ^***^p < 0.001.

Additionally, the interferon (IFN) response in infected A549 cells treated with procaine was reduced compared to the solvent-treated cells, which is potentially a further effect of the lag in endosomal release and RNA synthesis. We measured the mRNA expression of IFNβ, IFNλ1, IFNλ2/3, MxA (myxovirus resistance protein A) and IP-10 (IFN-gamma induced protein 10 kD) at 6 h p.i. and found their expression to be reduced by around 50% in PC and PHCl-treated infected cells compared to untreated infected cells (Figure 8). There was nonetheless a strong induction of the interferon response in all infected cells compared to the uninfected control cells.

### 3.5 PC and PHCl do not affect viral NA or HA activity

**Figure 9:**
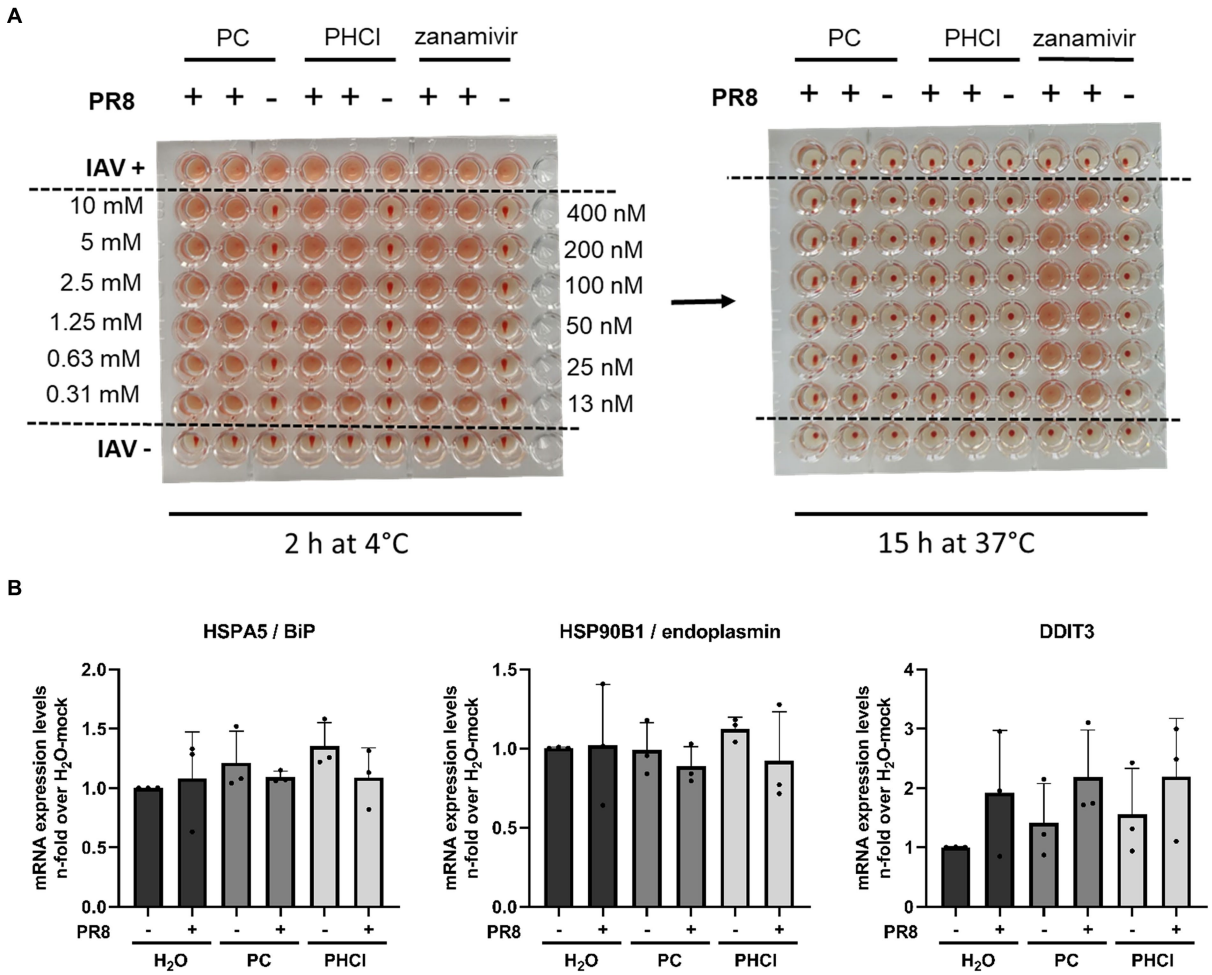
PC and PHCl treatment do not inhibit IAV HA and NA activity and do not cause an ER stress response. **(A)** Human erythrocytes were incubated with IAV PR8 as well as increasing concentrations of PC, PHCl (0.3 mM–10 mM), or zanamivir (12.5 nM–400 nM) in 96-well plates at 4°C for 2 h and hemagglutination of the erythrocytes was assessed at this time **(A, left picture)**. The plates were then incubated at 37°C for around 15 h over night and hemagglutination of the individual wells was assessed again to evaluate neuraminidase activity **(A, right picture)**. **(B)** A549 cells were infected with IAV PR8 at an MOI of 5 for 30 min and then treated with 2.5 mM PC or PHCl until 6 h p.i. Cells were washed and then lysed for RNA extraction. The relative amount of mRNA for the indicated ER-stress markers was assessed by qRT-PCR using GAPDH levels for normalization. RNA expression of untreated uninfected cells was arbitrarily set to 1. The mean + SD of three independent experiments with two biological replicates is depicted.

Since PC and PHCl treatment of IAV infection in A549 cells only caused transient effects on IAV RNA and protein synthesis as well as virus endosome fusion, but nonetheless inhibited progeny virus titers even when added late in the replication cycle, an additional effect of the substances towards the end of the replication cycle appears to exist. A possible late action of IAV inhibitors is inhibition of the viral NA activity. We used human erythrocytes to investigate whether PC and PHCl affected the activity of HA or NA (Figure 9A). The erythrocytes were incubated with IAV PR8 and a range of concentrations of PC, PHCl or zanamivir (a commercially available neuraminidase inhibitor) at 4°C for 2 h. In these conditions viral HA, which unlike NA activity does not require higher temperatures, can cause hemagglutination of the erythrocytes. None of the substances affected hemagglutination by the virus. The plates were then incubated at 37°C over night to allow for NA activity, which reverses the hemagglutination caused before. As would be expected zanamivir inhibited the reversal of hemagglutination. PC and PHCl even in concentrations up to 10 mM had no effect on NA activity.

The translation of viral proteins HA, NA and M2 occurs at the ER. The proteins translocate into the ER and are transported to the plasma membrane via the Golgi apparatus for budding. We used qRT-PCR to measure the mRNA expression of heat shock protein (HSP) A5/binding immunoglobulin protein (HSPA5/BiP), HSP90B1/endoplasmin and DDIT3 (DNA damage inducible transcript 3), two ER chaperone proteins and a transcription factor, respectively, that are upregulated in response to ER stress. None of the three ER stress markers were upregulated 6 h p.i. independent of procaine or solvent treatment (Figure 9B), which suggests that PC and PHCl do not affect trafficking of viral proteins through the ER, as the accumulation of viral protein in the ER would likely lead to the induction of ER stress.

### 3.6 PC and PHCl inhibit PLA_2_ from A549 but not MDCK cells

**Figure 10:**
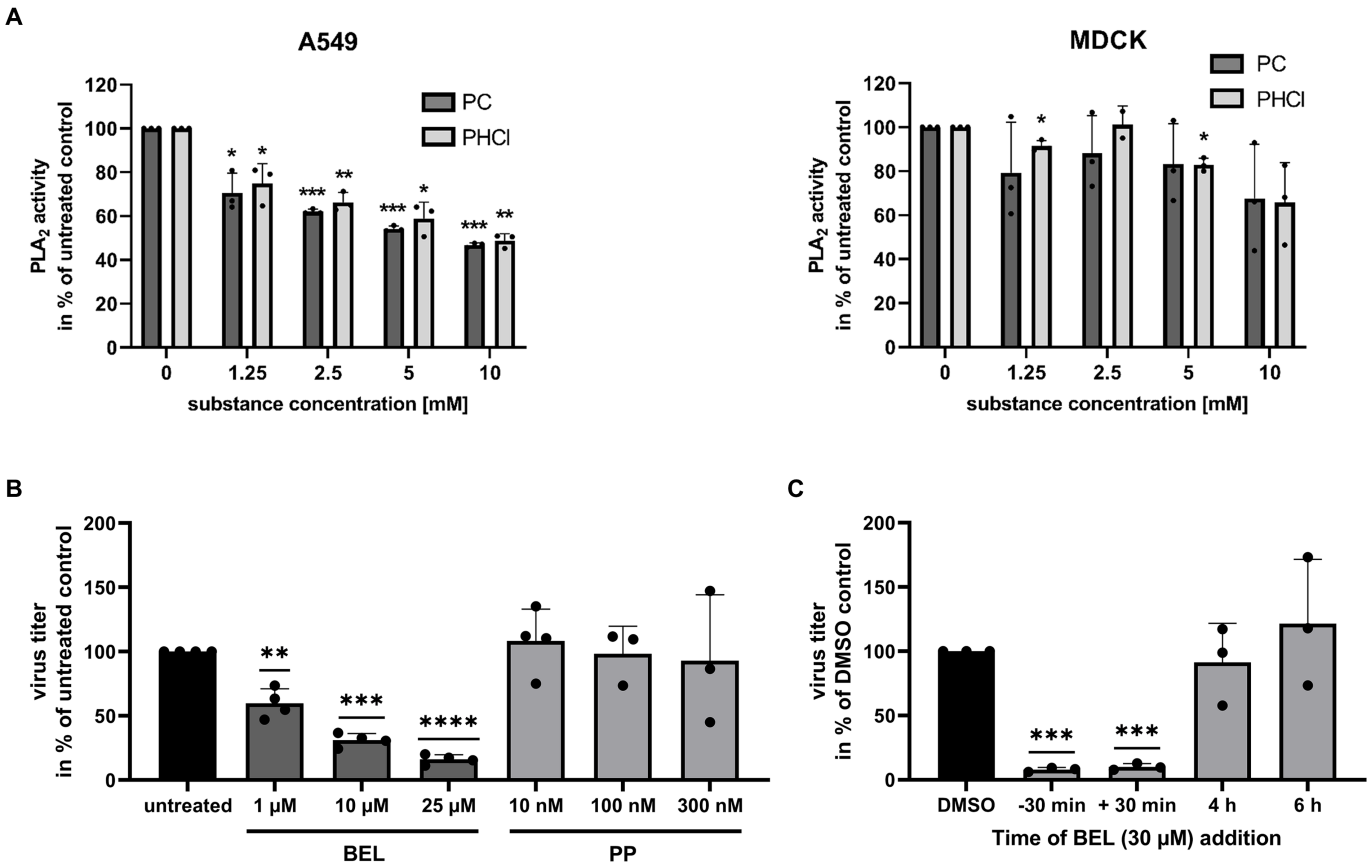
PC and PHCl inhibit PLA_2_ from A549 but not MDCK cells. **(A)** A549 and MDCK cells were lysed for 30 min on ice using distilled water. The activity of cellular PLA_2_ in the lysate was assessed in the presence of the indicated concentrations of PC and PHCl. PLA_2_ activity of untreated samples was arbitrarily set to 100%. **(B)** Calu-3 cells were infected with IAV PR8 at an MOI of 0.1 for 30 min and then treated with the indicated concentrations of bromoenol lacton (BEL), pyrrophenone (PP) or left untreated until 24 h p.i. Virus titers were determined by standard plaque assay and the titer in the solvent-treated samples was arbitrarily set to 100%. **(C)** Calu-3 cells were treated (−30 min) with 30 μM bromoenol lacton (BEL) or left untreated for 30 min prior to infection. Cells were then infected with PR8 at an MOI of 1 for 30 min. Afterwards cells were treated with 30 μM BEL or solvent control (DMSO) immediately (−30 min, 30 min and DMSO) or at the indicated times (without removing the media). **(A–C)** Statistical significance was determined by one sample *t*-test comparing to 100. ^*^p < 0.05, ^**^p < 0.01, ^***^p < 0.001, and ^****^p < 0.0001.

Procaine has also been reported to act on PLA_2_, a group of enzymes responsible for lysing membrane phospholipids producing a lysophospholipid and a free fatty acid. There are several subtypes of PLA_2_ and there are varying reports on the effects of procaine on these enzymes (Waite and Sisson, 1972; Hendrickson and van Dam-Mieras, 1976). We used a commercially available fluorescent substrate to investigate the effects of procaine on intracellular PLA_2_ from A549 and MDCK cells. We lysed the cells by treatment with distilled water to gain a PLA_2_ containing sample for the assay. Notably, PC and PHCl treatment did inhibit the activity of PLA_2_ from A549 cells by 30 to 40% in this assay at the concentrations we used in cell culture (Figure 10A, left panel). PLA_2_ activity was further reduced at 5 and 10 mM. We previously published results showing that PC and PHCl inhibit the activity of PLA_2_ from Calu-3 cells in the same way (Häring et al., 2023). PLA_2_ from MDCK cells however was not affected by PC or PHCl treatment here (Figure 10A, right panel).

To confirm whether inhibition of PLA_2_ has antiviral effects in our cells we used the specific inhibitors, bromoenol lacton (BEL) and pyrrophenone (PP) which inhibit calcium independent PLA_2_ (iPLA_2_) and cytosolic PLA2 (cPLA_2_), respectively. Both of these PLA_2_ enzymes should be present intracellularly. Treatment of PR8-infected Calu-3 cells with BEL for 24 h p.i. caused a concentration-dependent reduction in virus titers. They were reduced around 40, 60 and 75% with 1 μM, 10 μM and 25 μM BEL, respectively. PP in concentrations of 10 nM, 100 nM and 300 nM did not affect virus titers. We next conducted a 9 h time of addition experiment similar to the experiment shown in Figure 4 using BEL to investigate how iPLA_2_ inhibition effects viral replication at different times during the replication cycle (Figure 10C). We infected Calu-3 cells with 1 MOI of PR8 and added 30 μM BEL starting 30 min prior to infection, directly after infection, 4 h p.i. or 6 h p.i. and measured virus titers at 9 h p.i. Addition of BEL directly after the initial infection (with or without pre-incubation) caused a strong reduction in virus titers, while addition at 4 h or later had no effect.

Overall, PC and PHCl exhibit an antiviral effect in all three cell lines used here and affect several stages of the replication cycle at early and late times after infection. However, the specific steps of the replication cycle that are affected differ between the cell lines used.

## 4 Discussion

Over the past decades, local anesthetics in general and procaine specifically have been investigated for several purposes not related to analgesia, such as antioxidant, anti-inflammatory and anticancer effects (Gradinaru et al., 2021). Studies with procaine on HSV, WNV, VSV and Junin virus conducted in the 1970s, 80s and 90s already showed inhibitory effects of procaine on these viruses (Fuchs and Levanon, 1978; Bussereau and Genty, 1980; Castilla et al., 1994). Our own studies recently demonstrated that PC and PHCl treatment inhibit the replication of SARS-CoV-2 in Calu-3 and Vero-76 cells. We observed an inhibition of viral RNA replication as well as viral release from SARS-CoV-2-infected cells (Häring et al., 2023). In the present study we show that PC and PHCl treatment result in inhibition of IAV replication (Figures 2, 3). Previously, procaine treatment was reported to affect the attachment or entry of HSV and WNV into the host cell when cells were treated prior to infection (Fuchs and Levanon, 1978). We did not observe an effect of pre-incubation with PC or PHCl on IAV uptake (Supplementary Figure S4). Nonetheless, independent of the cell line used, virus titers were decreased when treatment was started 30 min after viral internalization (Figure 4). Part of this antiviral effect is likely due to the delayed release of viral material from the endosome that we observed (Figure 7). This process is highly pH dependent, requiring acidification of the endosome and subsequent acidification of the viral particles via the M2 ion channel. Local anesthetics exert their primary function of anesthesia by blocking voltage-dependent sodium channels on nerve fibers and procaine has also been found to act on Ca^2+^- and K^+^-channels (Zahradníková and Palade, 1993; Huang et al., 1999; Wang et al., 2013). The delay in IAV RNA release from the endosome could in theory be due to direct inhibition of channels involved in the acidification of the endosome or the viral M2 ion channel. However, the inhibition of virus endosome fusion could also be explained by a more indirect mechanism. Procaine, the active component in both PC and PHCl, is a weak base. Drugs that are weak bases, including local anesthetics, are known to accumulate in endocytic compartments. These drugs can diffuse into the organelle in the uncharged state and are then protonated due to the low pH in the endosome and cannot diffuse back out (Aki et al., 2012; Marceau et al., 2014). This process is known as cation-trapping. Accordingly, previous investigations into the effects of different amines including procaine on endosomal pH and VSV release from the endosome demonstrated 50% inhibition of virus endosome fusion at 3.3 mM procaine, a concentration around 15-times higher than that required of the local anesthetic lidocaine and around 150-times higher than that required of chloroquine (Miller and Lenard, 1981). The partial or transient inhibition of virus endosome fusion we observed with 2.5 mM PC or PHCl treatment is in line with reported results. Cation-trapping is also known to cause osmotic vacuolization (Marceau et al., 2014), which we also observed here starting from concentrations of about 6 mM in Calu-3 and A549 cells and about 3 mM in MDCK cells. It is not entirely understood if vacuolization is harmful to the cells (Aki et al., 2012), although excessive vacuolization is unlikely to be beneficial.

PC or PHCl treatment affects viral RNA replication and subsequent protein synthesis differently in the cell lines investigated here. Even though continuous PC or PHCl treatment started 30 min p.i. both viral RNA and protein synthesis were only transiently inhibited in A549 cells and recovered towards the end of the replication cycle (Figures 5, 6). In contrast, RNA levels remained decreased compared to the control from 4 h onward in Calu-3 and MDCK cells. At least part of the effect on viral RNA replication observed is likely due to the described inhibition of virus release from the endosome. However, the fact that the inhibitory effect is much stronger in Calu-3 and MDCK cells suggests that procaine exerts a further more direct effect on viral replication in these cells. Local anesthetics such as procaine have been described to interact with biological membranes, changing their organization and fluidity and this has been suggested as an underlying mechanism of their effects on various channels (Tsuchiya and Mizogami, 2013; Krogman et al., 2023). The wide range of proteins that could potentially be affected by procaine treatment through such a mechanism makes it difficult to identify a specific target, responsible for the procaine-mediated antiviral effect we observed.

A549 and, to a lesser extent, Calu-3 cells showed diminished virus titers even when PC or PHCl was added late in infection (Figures 4A,B). This suggests that progeny virus release was influenced by procaine. Virus titers from MDCK cells were only reduced if the substances were added right after infection (Figure 4C). Since both, the expression of ER stress markers as well as the activity of viral HA and NA was unaffected by treatment (Figure 9), we concluded that improper processing of viral proteins in the ER or inhibited release from the cell surface were not the cause of this late effect. However, both PC and PHCl inhibited the activity of PLA_2_ enzymes of A549 cells but not MDCK cells (Figure 10A). We have previously also shown that PC and PHCl are able to inhibit the activity of PLA_2_ from Calu-3 cells (Häring et al., 2023). PLA_2_ enzymes hydrolyze membrane phospholipids at the sn-2 (stereospecifically numbered 2) position of one of their fatty acid chains. This alters the shape of the resulting lysophospholipid, which can play an important role in membrane remodeling activity (Brown et al., 2003). There are several different groups of PLA_2_ enzymes in humans and animals that have been described in detail (Schaloske and Dennis, 2006). The main types of PLA_2_ enzymes are secreted PLA_2_ (sPLA_2_), cPLA_2_, iPLA_2_, PAF acetylhydrolases (PAF-AH) and lysosomal PLA_2_. The EnzChek^™^ PLA_2_ assay kit detects the activity of any phospholipase capable of the hydrolysis of phospholipids at the sn-2 position. The way in which the cells were lysed for the assay excludes any of the PLA_2_ enzymes that are extracellular, specifically all sPLA_2_ and one subgroup of PAF-HA, from contributing to the assay. It has previously been shown that specific inhibition of cPLA_2_ does not affect the replication of IAV (Müller et al., 2018) while inhibition of iPLA_2_ does, potentially by influencing membrane bending and inhibiting virus budding (Nakano et al., 2009). We could confirm that inhibition of iPLA_2_ using BEL leads to a dose-dependent reduction in viral titers while inhibition of cPLA_2_ using PP in concentrations of up to 300 nM does not (Figure 10B). PP inhibits cPLA_2_ with an IC_50_ of 4.2 nM in a cell free system and inhibits the release of arachidonic acid (a common product of phospholipid hydrolysis by PLA_2_ enzymes) with an IC_50_ of 190 nM in human blood (Seno et al., 2001). Previous research indicated that inhibition of iPLA_2_ affected virus budding, raising the hypothesis that PLA_2_ inhibition may be involved in the effects late in the replication cycle in A549 and Calu-3 cells. However, addition of BEL 4 h or 6 h after infection had no effect on viral titers in our experiments (Figure 10C). This suggests that the antiviral effect of PC and PHCl at late stages is not due to iPLA_2_ inhibition. However, inhibition of iPLA_2_ or other PLA_2_ subtypes could contribute to the overall antiviral effect of PC and PHCl.

Both Calu-3 and A549 cells are adenocarcinoma-derived human lung epithelial cells and therefore could be considered a better model for infection in humans than MDCK cells, which are canine kidney epithelial cells. However, the effects of PC and PHCl during the replication cycle vary even between Calu-3 and A549 cells. In future primary lung epithelial cells, more complex cell culture systems or *in vivo* models could be used to further investigate the effects of procaine on viral replication. *In vivo* experiments would further aid in determining the safety of PC as an antiviral treatment. We show that PC is 50% effective at around 0.5 mM (Figure 2) and starts to cause potentially harmful changes to cells at around 10-times that concentration (Figure 1; Supplementary Figure S3). PC could be applied by inhalation in *in vivo* experiments and this might improve the relationship of required dose and potential systemic toxicity. Nebulization of local anesthetics to achieve topical anesthesia in the respiratory tract has been described since the 1940s and has since been used for endoscopic procedures and investigated for its use in asthma and SARS-CoV-2 infection (Miller et al., 1949; Slaton et al., 2013; Welter et al., 2023). Application of the local anesthetic lidocaine via inhalation has been shown to cause lower plasma concentrations compared to other applications, with subsequently reduced toxicity (Labedzki et al., 1990; Slaton et al., 2013). Future investigations would, however, still need to clarify the doses that could be reached safely by inhalation without direct toxicity to the lung.

PC and PHCl treatment also led to reduced IAV-induced mRNA expression of the type I and type III IFNs IFN-β and IFN-λ1, -2 and -3 and the IFN-stimulated MxA and IP-10 in A549 cells 6 h p.i. (Figure 8). There has been previous descriptions of a dependence of the strength of IFN-λ1 induction on cellular MOI within the first 8 h p.i. in A549 cells (Martin et al., 2020). The reduction in IFN expression seen here is likely related to a reduced pathogen load.

We do not observe any clear differences in the antiviral activity and mechanism of PC and PHCl treatment, even though these two compounds exhibit different chemical properties such as different pH values in solution. Membrane penetration depends on the balance of the charged (active) and uncharged (transport) form of these substances, which is also improved with PC compared to PHCl. However, the differences in the chemical properties of these substances do not seem to have any relevant effects in simple cell culture models such as the ones used here. Nonetheless, the different properties of PC and PHCl probably play a larger role in a more complex system.

Overall, we show that PC and PHCl affect viral replication *in vitro* at several points during the replication cycle. Although variations can be seen in the mechanism of action between the three cell lines, virus titers are reduced by a comparable amount in all of them in multi-cycle experiments.

## Data availability statement

The original contributions presented in the study are included in the article/Supplementary material, further inquiries can be directed to the corresponding author.

## Ethics statement

Ethical approval was not required for the studies involving humans because the used erythocytes were a commercially purchased approved blood product (Erythrocyte Concentrate SAGM Th-J; Institute for Clinical Transfusion Medicine Jena gGmbH) PEI.H04332.01.1. The studies were conducted in accordance with the local legislation and institutional requirements. The human samples used in this study were acquired from the Institute for Clinical Transfusion Medicine Jena gGmbH. Written informed consent to participate in this study was not required from the participants or the participants’ legal guardians/next of kin in accordance with the national legislation and the institutional requirements.

## Author contributions

CH: Conceptualization, Formal analysis, Investigation, Visualization, Writing – original draft, Writing – review & editing. JS: Formal analysis, Investigation, Writing – review & editing. JJ: Formal analysis, Investigation, Writing – review & editing. BL: Funding acquisition, Writing – review & editing. AH: Writing – review & editing. BE: Formal analysis, Funding acquisition, Investigation, Resources, Writing – review & editing. CE: Conceptualization, Funding acquisition, Project administration, Resources, Supervision, Writing – review & editing.
