# Supplementary material for: ProcCluster® and procaine hydrochloride inhibit the replication of influenza A virus in vitro
Source: Front Microbiol. 2024 Aug 14;15:1422651. doi: 10.3389/fmicb.2024.1422651 (PMC11350405; doi:10.3389/fmicb.2024.1422651)
Supplement: Supplementary file 1 [file Data_Sheet_1.PDF]

A

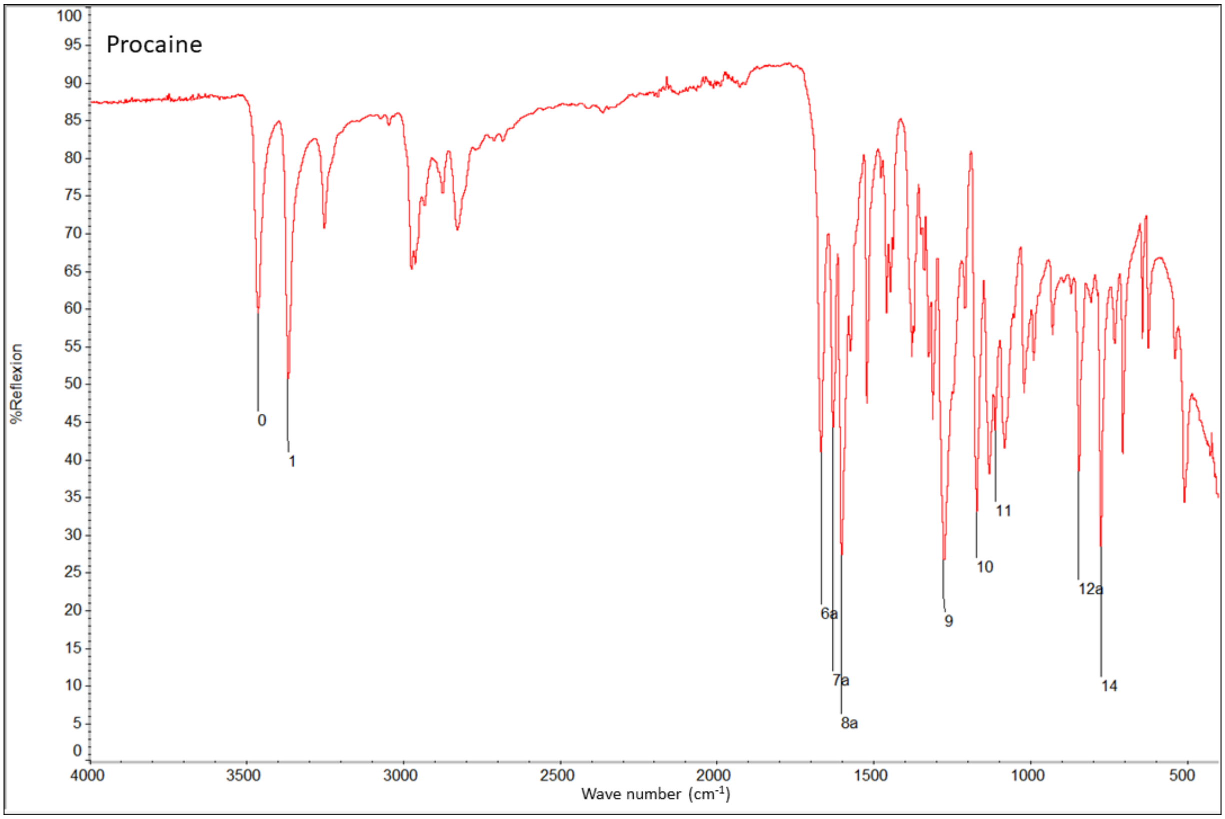

Wave number (cm-1)

B

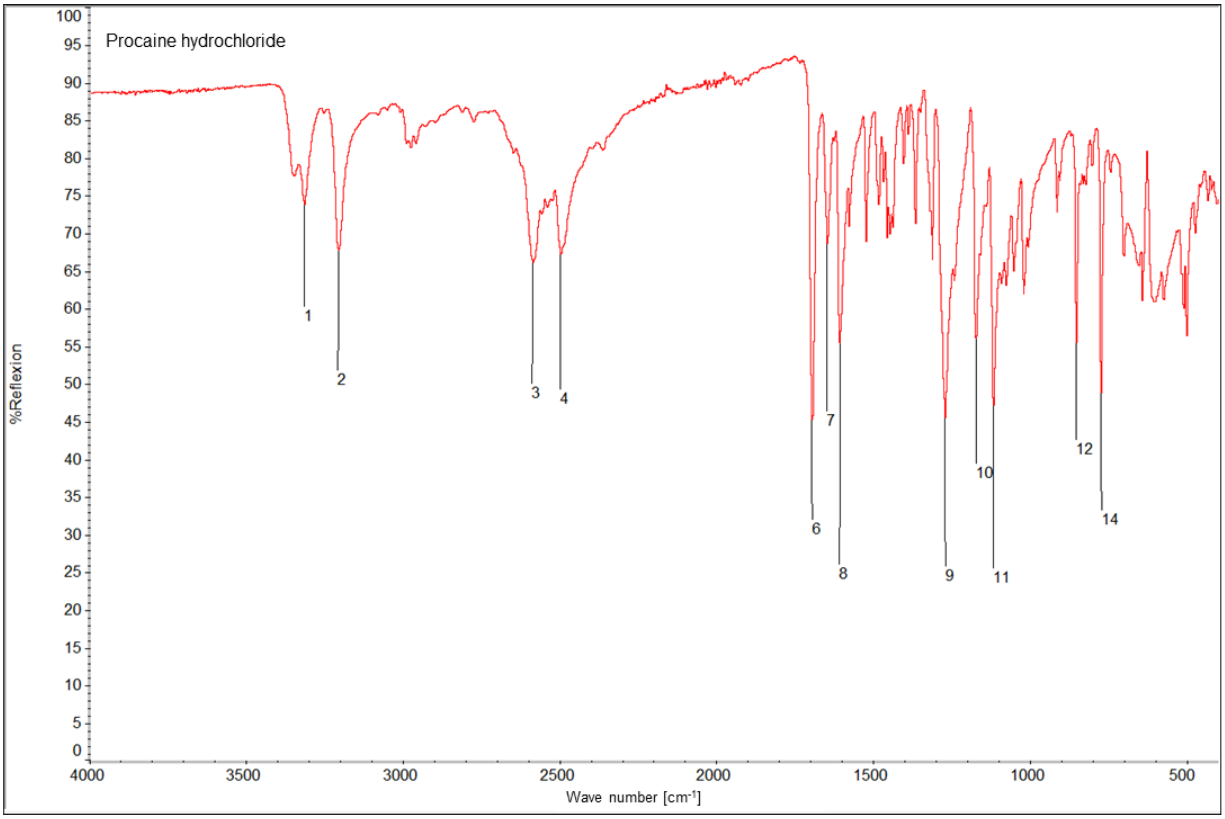

C

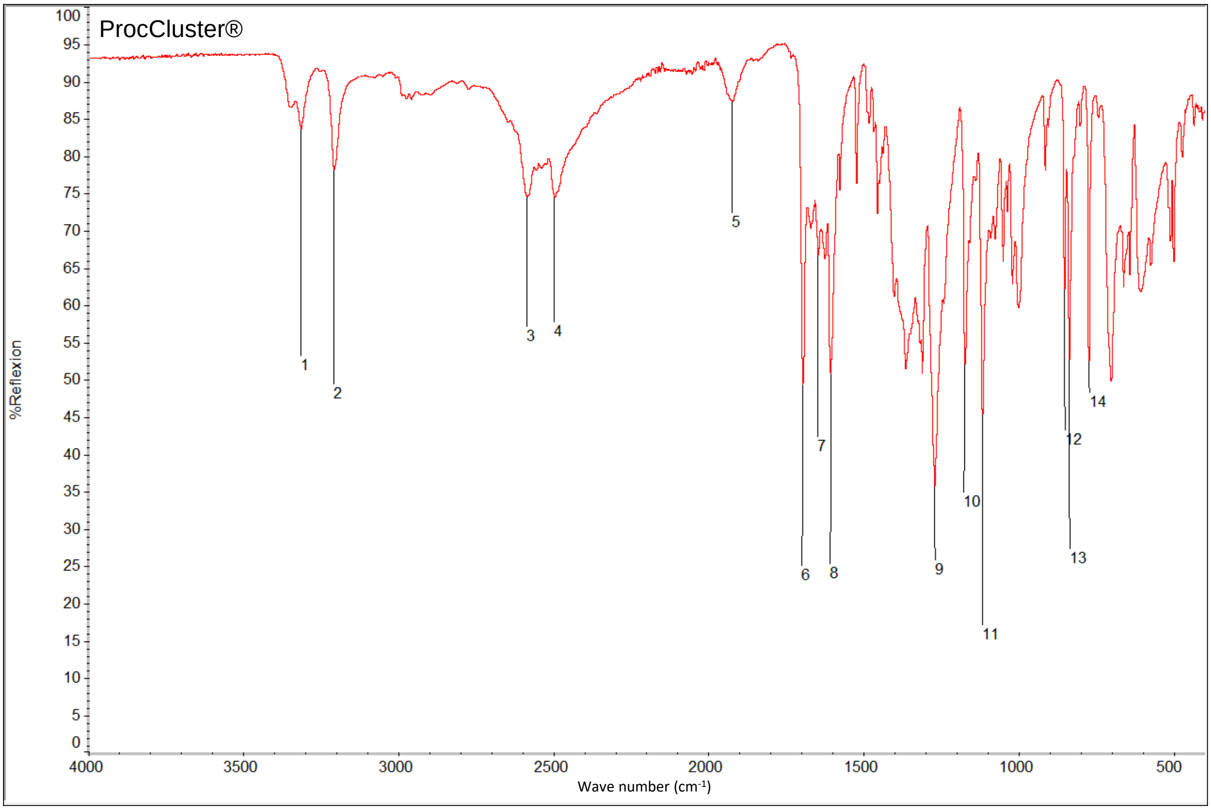

D

#	**Allocation in the molecule / Vibration**	Procaine ũ [cm-1]	PHCl ũ [cm-1]	ProcCluster ũ [cm-1]
0	2 bands, primary amine / N-H-valence	3462	—	—
1	2 bands, primary amine / N-H-valence	3365	3312	3312
2	ammonium salts / NR_3H^+-valence (by PHCl and ProcCluster®)	—	3203	3205
3	ammonium salts	—	2583	2583
4	ammonium salts	—	2493	2493
5	hydrogen carbonate by ammonium salts	—	—	1920
6	ester / carbonyl vibration (C=O)	—	1691	1692
6a	ester / carbonyl vibration (C=O)	1665	—	—
7	primary amine / NH_2-deformation	—	1643	1644
7a	primary amine / NH_2-deformation	1625	—	—
8	aromatic compounds / ring vibration	—	1603	1603
8a	aromatic compounds / ring vibration	1599	—	—
9	aromatic ester / –C–O–C-valence	1272	1267	1267
10	amin / C–N–valence	1168	1169	1170
11	C–O–vibration	1108	1113	1113
12	1,4-substituted aromatic compounds / =C-H-deformation	—	848	849
12a	1,4-substituted aromatic compounds / =C-H-deformation	841	—	—
13	hydrogen carbonate / HCO_3^-	—	—	833
14		772	770	770

E

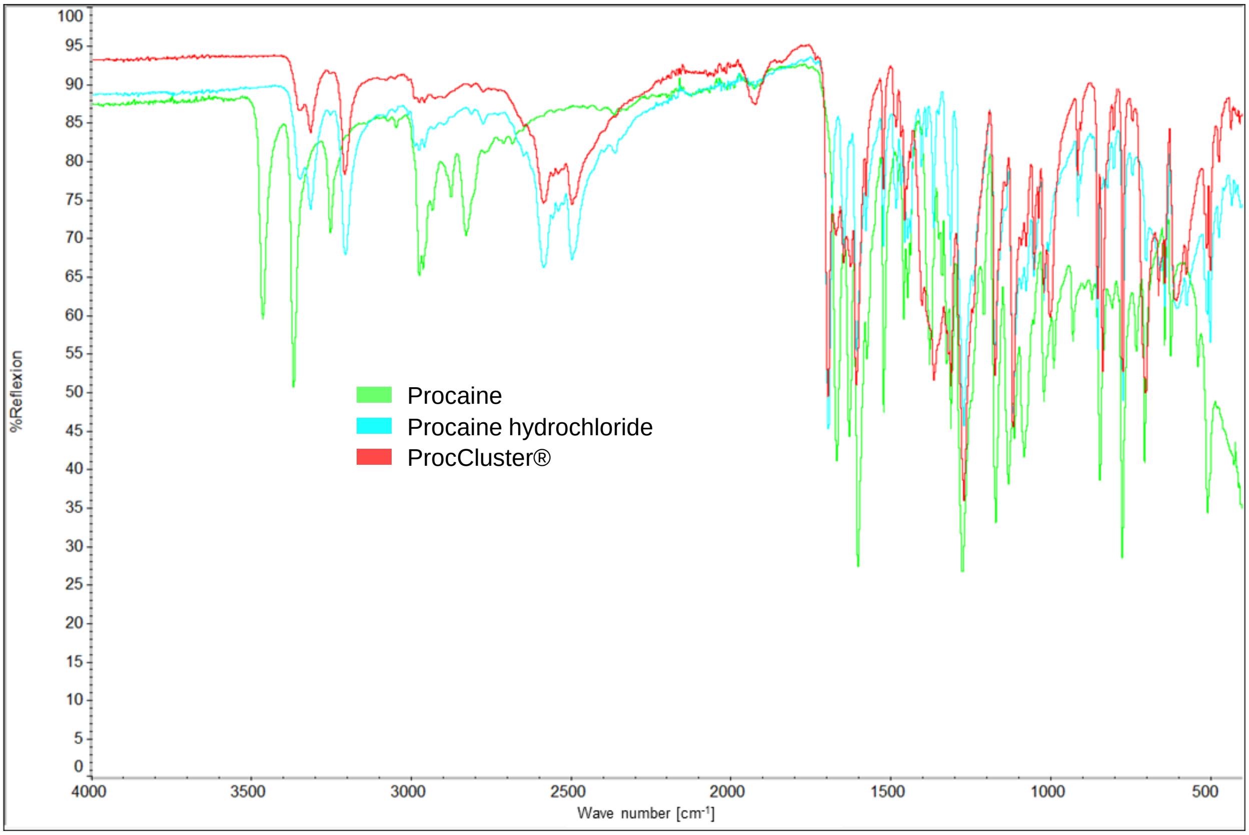

Supplementary Figure S1: IR-spectra of procaine, procaine hydrochloride and ProcCluster®. Attenuated total reflection IR-spectroscopy was carried out on samples of (**A**) procaine, (**B**) procaine hydrochloride and (**C**) ProcCluster®. (**D**) Explanations for numbered sections. (**E**) Overlay of IR-spectra of all three substances.

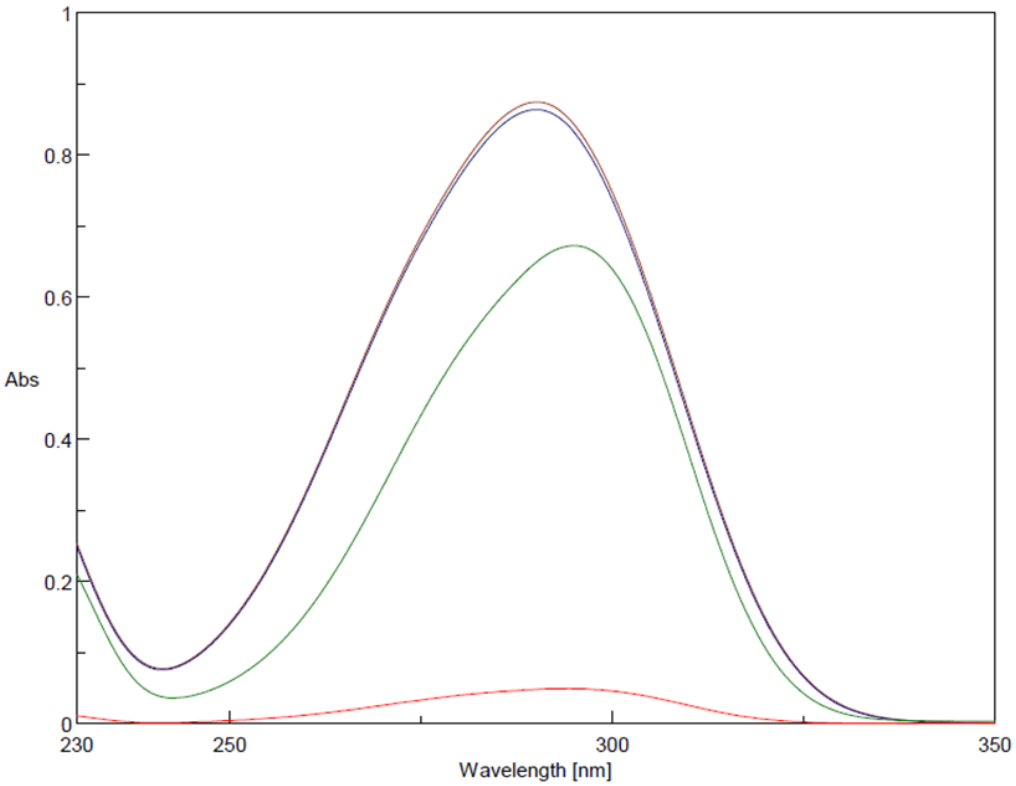

Supplementary Figure S2: Solubility of ProcCluster® and procaine hydrochloride in water and octanol. Procaine hydrochloride and ProcCluster® were added to water or octanol to a theoretical concentration of 0.046 mM. After 2 h, 250 µl of the solution were added to a new flask with 10 ml of the respective solvent (water or octanol) and the pictured UV/VIS spectrum was obtained. Red: Procaine hydrochloride in octanol; Brown: Procaine hydrochloride in water; Green: ProcCluster® in octanol; Blue: ProcCluster® in water.

A

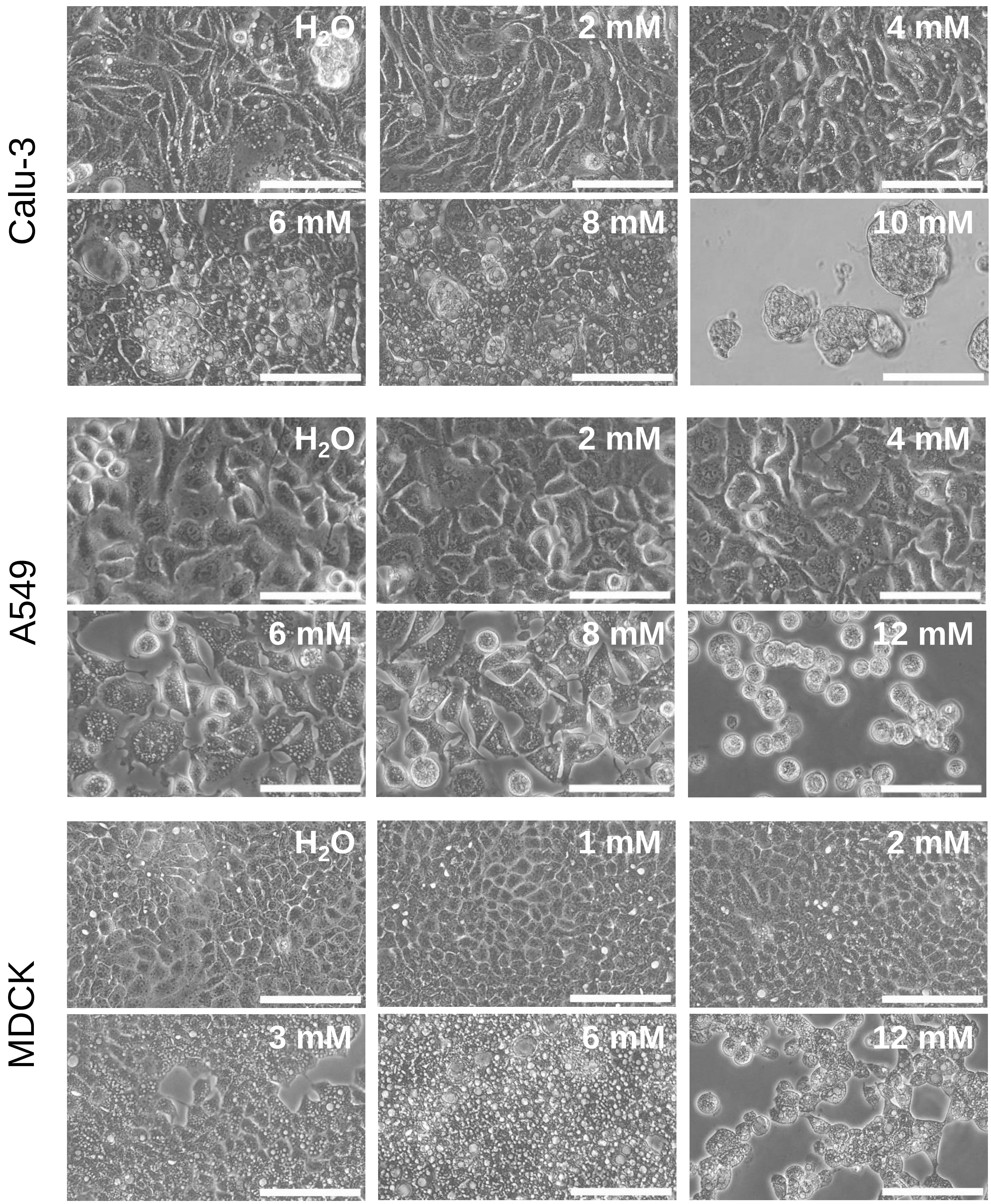
Calu-3
H2O
2 mM
4 mM
6 mM
8 mM
10 mM
A549
H2O
2 mM
4 mM
6 mM
8 mM
12 mM
MDCK
H2O
1 mM
2 mM
3 mM
6 mM
12 mM

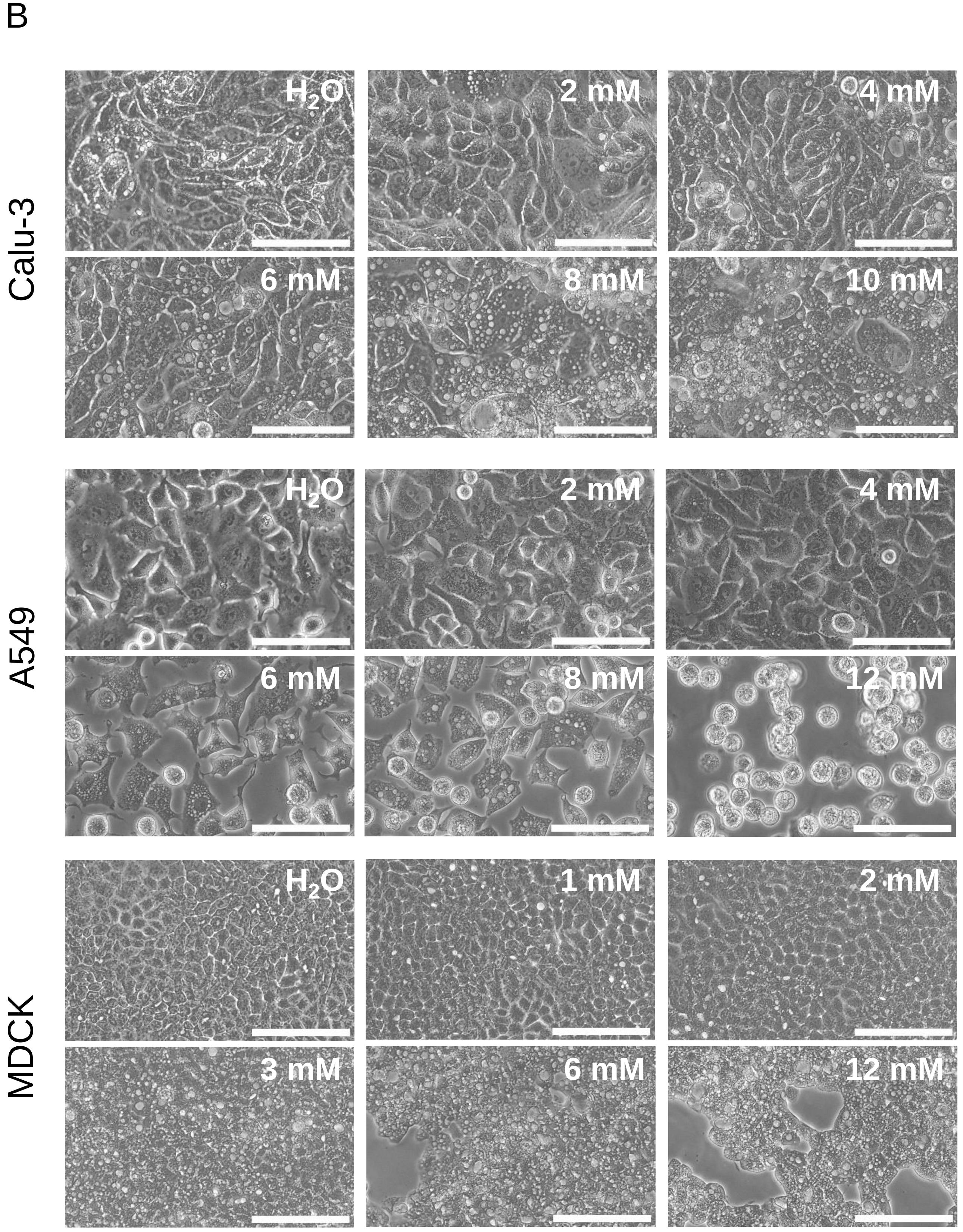

Supplementary Figure S3: High concentrations of PC and PHCl cause osmotic vacuolization. Calu-3 cells, A549 cells and MDCK cells were treated with the indicated concentrations of PC (**A**) or PHCl (**B**) in medium with 10% FCS and light microscopy pictures were taken after 24 h. Scale bar represents 100 µm.

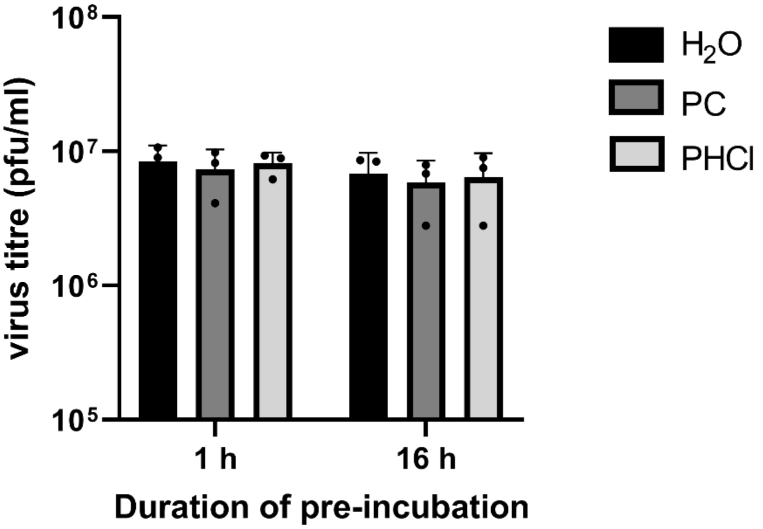

Supplementary Figure S4: Pre-incubation with PC or PHCl prior to infection does not affect virus titers. Calu-3 cells were incubated with PC or PHCl or solvent control (H_2O) for 16 h or 1 h prior to infection. The cells were then infected with 1 MOI of A/Puerto Rico/8/34 and supernatants were collected at 9 h p.i. Viral titers were determined by standard plaque assay. The mean +SD of three independent experiments is depicted.
